# RNA-binding protein RBM3 intrinsically suppresses lung innate lymphoid cell activation and inflammation partially through CysLT1R

**DOI:** 10.1038/s41467-022-32176-5

**Published:** 2022-07-30

**Authors:** Jana H. Badrani, Allyssa N. Strohm, Lee Lacasa, Blake Civello, Kellen Cavagnero, Yung-An Huang, Michael Amadeo, Luay H. Naji, Sean J. Lund, Anthea Leng, Hyojoung Kim, Rachel E. Baum, Naseem Khorram, Monalisa Mondal, Grégory Seumois, Julie Pilotte, Peter W. Vanderklish, Heather M. McGee, Taylor A. Doherty

**Affiliations:** 1 Divison of Rheumatology, Allergy and Immunology, Department of Medicine, University of California San Diego, La Jolla, CA USA; 2 Veterans Affairs San Diego Health Care System, La Jolla, CA USA; 3 Department of Microbiology and Immunology, College of Medicine, Chang Gung University, Taoyuan, Taiwan; 4 La Jolla Institute, La Jolla, CA USA; 5 The Scripps Research Institute, La Jolla, CA USA; 6 NOMIS Center for Immunobiology and Microbial Pathogenesis, Salk Institute, La Jolla, CA USA; 7 Departments of Radiation Oncology and Immuno-Oncology, City of Hope, Duarte, CA USA; 8 Department of Molecular Medicine, La Jolla, CA USA

**Keywords:** Asthma, Innate lymphoid cells, Mucosal immunology

## Abstract

Innate lymphoid cells (ILC) promote lung inflammation in asthma through cytokine production. RNA-binding proteins (RBPs) are critical post-transcriptional regulators, although less is known about RBPs in ILC biology. Here, we demonstrate that RNA-binding motif 3 (RBM3) is highly expressed in lung ILCs and is further induced by alarmins TSLP and IL-33. *Rbm3*^*−/−*^ and *Rbm3*^*−/−*^*Rag2*^*−/−*^ mice exposed to asthma-associated *Alternaria* allergen develop enhanced eosinophilic lung inflammation and ILC activation. IL-33 stimulation studies in vivo and in vitro show that RBM3 suppressed lung ILC responses. Further, *Rbm3*^*−/−*^ ILCs from bone marrow chimeric mice display increased ILC cytokine production suggesting an ILC-intrinsic suppressive function of RBM3. RNA-sequencing of *Rbm3*^*−/−*^ lung ILCs demonstrates increased expression of type 2/17 cytokines and cysteinyl leukotriene 1 receptor (CysLT1R). Finally, *Rbm3*^*−/−*^*Cyslt1r*^*−/−*^ mice show dependence on CysLT1R for accumulation of ST2^+^IL-17^+^ ILCs. Thus, RBM3 intrinsically regulates lung ILCs during allergen-induced type 2 inflammation that is partially dependent on CysLT1R.

## Abstract (web summary)

The function of RNA binding proteins within innate lymphoid cells (ILC) has been partially characterised. Here the authors show that RBM3 functions to limit the type 2 immunity promoting activity of ILC2 partially through cysteinyl leukotriene 1 receptor.

## Introduction

Lung innate lymphoid cells (ILC) are critical players in inflammatory diseases including helminth infections, asthma, and pulmonary fibrosis^1–4^. Once activated by epithelial cytokines, such as TSLP, IL-33, IL-25, and lipid mediators including leukotrienes, group 2 innate lymphoid cells (ILC2) produce type 2 cytokines IL-4, IL-5, IL-9, and IL-13^5,6^. These cytokines are major contributors to the characteristics of type 2 asthma such as airway inflammation, hyperresponsiveness, and remodeling. IL-13 promotes mucus production and airway hyperresponsiveness, IL-4 regulates IgE synthesis and Th2 cell differentiation, and IL-5 controls the survival and activation of eosinophils^7,8^. In addition to the contribution of ILC2s in asthma, there is also evidence that IL-17A production by “inflammatory” iILC2s (or ILC2-17s) as well as ILC3s promote lung inflammatory responses in asthma models^2,9,10^. For example, cysteinyl leukotrienes induce IL-17A from ST2^+^ lung ILC2s and transferred IL-17^−/−^ ILC2s are less pathogenic during type 2 lung inflammation^10^. Since the discovery of ILCs, most studies have focused on mechanisms of ILC activation, and there are fewer reports that provide insights into suppression of ILC responses. Modulation of ILC2s may occur through individual cytokines^11,12^ and lipid mediators, and cell-cell contact pathways^13,14^. Interestingly, despite the known regulatory activity of microRNAs (miRNAs) in post-transcriptional repression, studies have demonstrated that miR-19 and miR-155 promote ILC2 activation and survival^15,16^. Overall, our understanding of mechanisms that broadly suppress both type 2 and 17 cytokine production in lung ILCs is limited.

Regulation of immune cell responses occurs through gene expression as well as post-transcriptional and post-translational pathways. RNA-binding proteins (RBPs) regulate cellular responses via stabilization or degradation of mRNA transcripts as well as effects on miRNA processing^17^. The RBP HuR stabilizes target mRNAs including *gata3* transcripts in CD4^+^ Th2 cells through biding to AU-rich element (ARE) sequences in the 3’UTR^18^. AREs are also present in Th2-cytokine transcripts including *Il4*, *Il5*, *Il13*, and therefore are potential posttranscriptional regulatory targets for RBPs^19^. Very recently, naïve ILC2s were shown to express the RBP tristetraprolin (TTP) which inhibits Th2 cytokine production and is downregulated after IL-33 stimulation^20^. Aside from these studies, how RBPs control ILC responses is not well explored and likely represents an important level of ILC regulation in inflammatory diseases.

In this work, we perform RNA sequencing of purified lung ILC subsets from fungal allergen-challenged mice based on CD127 and ST2^21^ and assess levels of RBP transcripts in ILC subsets. The *Rbm3* transcript which encodes RNA-binding motif 3 (RBM3) is one of the most highly expressed RBPs in ILCs, second to *Zfp36* (encodes TTP)^20^, and with a higher expression level than *Elavl1*, which encodes for HuR. RBM3 is a cold shock protein that has been demonstrated to enhance the stability and translation of mRNAs for COX-2, IL-8, and VEGF^22^. Further, RBM3 interacts with microRNAs miR-142–5p and miR-143, temperature-sensitive microRNAs implicated in the fever response^23^, as well as with the RBP HuR^22^. Using fungal allergen-driven and IL-33-driven lung inflammation models with *Rbm3*^*−/−*^, *Rbm3*^*−/−*^*Rag2*^*−/−*^*, and Rbm3*^*−/−*^*Cyslt1r*^*−/−*^ mice, as well as in vitro studies, mixed bone marrow chimera studies, and transcriptomic analysis, we show that RBM3 negatively regulates lung ILC type 2 and 17 cytokine responses that are partially dependent on CysLT1R signaling. Contrary to previous reports showing stabilization of cytokine transcripts by RBM3, the data presented herein demonstrates that RBM3 suppresses ILC2 activation in the lung.

## Results

### RBM3 is highly expressed among RNA-binding protein mRNAs in activated lung ILCs

**Fig. 1:**
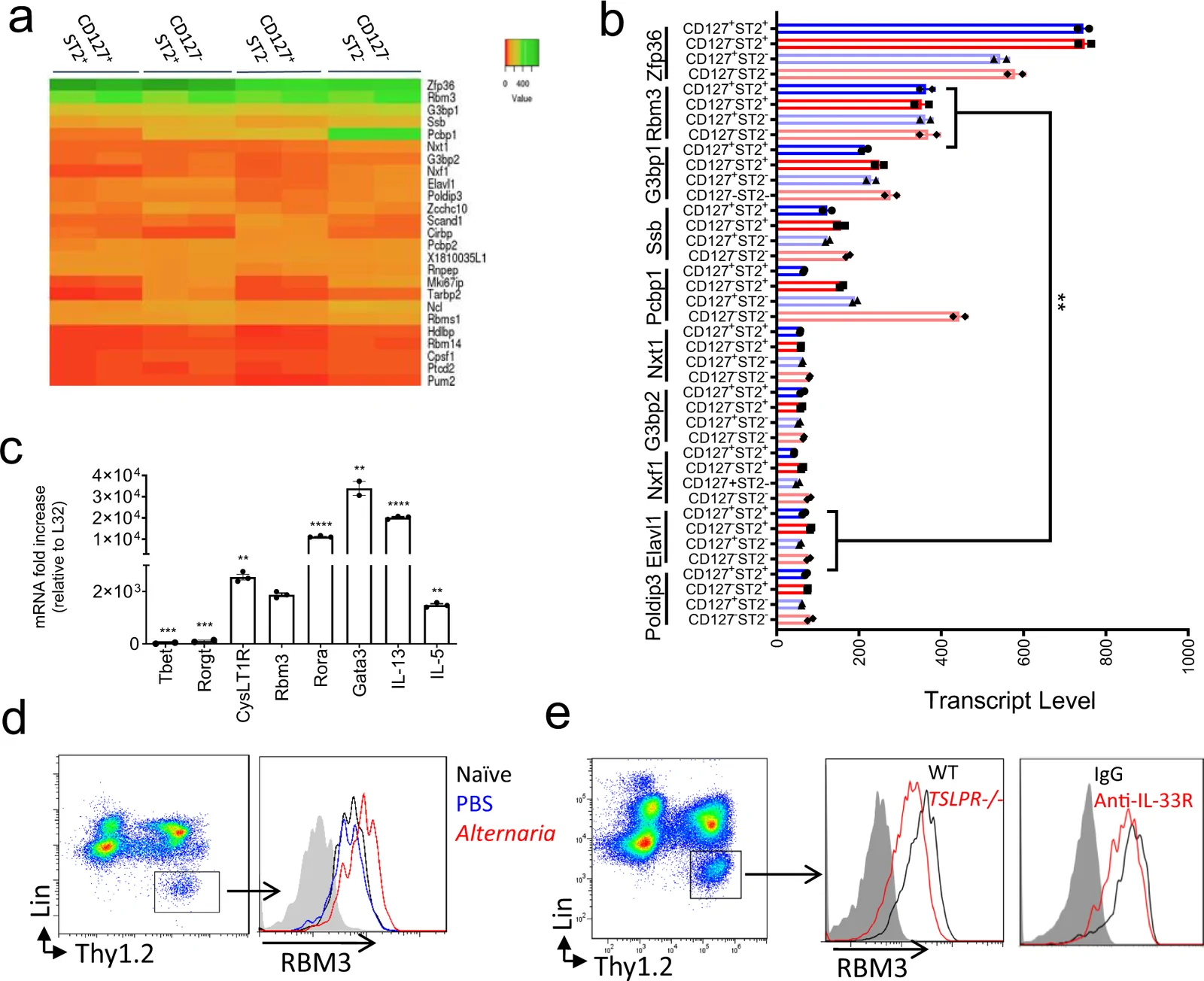
RNA-binding protein RBM3 is highly expressed by lung ILCs during fungal allergen exposure and is regulated by IL-33 and TSLP. **a** Wild-type mice were challenged 4 times with 50 µg *Alternaria* over the span of 10 days and four ILC subsets (CD127^+^ST2^+^, CD127^−^ST2^+^, CD127^+^ST2^−^, CD127^−^ST2^−^) were sorted and collected for RNA-seq. Heatmap showing the relative expression of the top 25 most highly expressed RBPs in the four ILC subtypes. RBPs involved in the cell cycle or splicing were excluded. Statistics were performed with Centroid linkage and Manhattan clustering. **b** Absolute transcript levels of the top 10 RBPs in the four ILC subtypes. Statistics were performed with an unpaired t-test, two-tailed. Data representative of 2 samples (*p* < 0.01). **c** RBM3 mRNA levels of ILC2s in comparison to several traditional ILC2 mRNA (IL-5, IL-13, Gata-3, Rora, and CysLT1R) and non-ILC2 mRNAs (Rorgt and Tbet). ILC2s were from *Alternaria* challenged mice. Data are from triplicate samples and analyzed with an unpaired t-test, two-tailed. **d** RBM3 expression on ILC2s from naive, PBS challenged, or *Alternaria* challenged lung. Black = Naive, Blue = PBS challenged, Red = *Alternaria* challenged (24 h). **e** RBM3 expression on lung ILC2s of *Tslpr*^*−/−*^ mice and mice treated with anti-IL-33R antibody. Data representative of at least 4 mice (paired) per group. Mice were challenged with *Alternaria* four times. Grey = isotype control, Black = Wild-Type or IgG treated, Red = *Tslpr*^*−/−*^ or anti^*-*^IL^*-*^33R treated. *p < 0.05, **p < 0.01, ***p < 0.001. Data are presented as mean values+/*−* SEM.

ILC subtypes can be defined by expression of CD127 (IL-7R) and ST2 (IL-33R) and we recently showed that single negative or double negative “unconventional” subpopulations for these markers include heterogenous ILC populations that express GATA-3 and produce type 2 cytokines^21^. Upon airway challenge with the asthma-associated fungal allergen *Alternaria alternata*, all four Thy1.2^+^ subpopulations (CD127^+^ST2^+^, CD127^+^ST2^−^, CD127^−^ST2^+^, and CD127^−^ST2^−^) are activated. Lin^−^Thy1.2^+^ lymphocytes from WT mice challenged over 10 days with 50 µg *Alternaria* were FACS purified based on expression of CD127 and ST2 (Supplementary Fig. 1a). Next, we performed RNA-Seq analysis and detected 207 RNA-binding protein (RBPs) transcripts expressed by the ILC subpopulations (Supplementary Fig. 1b). Of the top 25 differentially expressed RBPs (Fig. 1a), *Zfp36*, which encodes for tristetraprolin (TTP), was the most highly expressed RBP. *Zfp36-/-* mice develop spontaneous prominent inflammation and severe autoimmune disease^24^, and a very recent report demonstrates that TTP regulates ILC2 homeostasis^20^. After *Zfp36, Rbm3* was the next highest RBP with transcript levels increased over *Elavl1*, which encodes for HuR (Fig. 1b) which has been shown to promote GATA-3 expression and type 2 cytokines in T cells^18,25^. Since we were interested in RBP function using in vivo asthma models, we focused on regulation by RBM3 given that *Rbm3*^*−/−*^ mice are viable and not known to spontaneously have immune disease^25,26^. Sorted Thy1.2^+^ lung ILCs from *Alternaria*-challenged mice expressed *Rbm3* mRNA at levels on par with *Il5* mRNA though less than *Gata3*, *Rora*, and *Il13* (Fig. 1c). qPCR analysis of the four ILC populations based on ST2 and CD127 showed that *Rbm3* expression was highest in ST2^+^ ILCs that also more highly expressed *Cysltr1*, *Il5*, *Il13*, and *Il17* (Supplementary Fig. 2). Thus, of 207 RBP transcripts from in vivo activated lung ILCs, *Rbm3* was expressed at levels comparable to molecules involved in ILC function and thus is a candidate protein that might regulate ILC function and lung inflammation.

### Alternaria, IL-33, and TSLP increase lung and ILC RBM3 expression

To ascertain whether RBM3 is induced during type 2 inflammation, *Alternaria*-challenged mice were assessed for RBM3 levels by immunostaining. Increased expression of RBM3 was detected by immunofluorescence in *Alternaria*-challenged lungs compared to naïve control lungs (Supplementary Fig. 3a). RBM3 expression was visualized in epithelial as well as subepithelial cells which was further induced by challenge with *Alternaria*. As ILCs regulate innate lung responses *to Alternaria*^26^, we investigated changes in RBM3 levels in lung ILCs. Lin^−^Thy1.2^+^ ILCs from the lungs of challenged mice demonstrated increased RBM3 expression by intracellular flow cytometry when compared to naïve and PBS-challenged controls (Fig. 1d). Interestingly, eosinophils and macrophages analyzed from challenged mice did not show increases in RBM3 expression (Supplementary Fig. 3b).

As IL-33 and TSLP are critical epithelial cytokines that regulate ILC2 responses, we assessed levels of RBM3 in *Tslpr*^*−/−*^ mice and WT mice treated with IL-33 blocking antibody. RBM3 expression in *Tslpr*^*−/−*^ mice and WT mice receiving anti-IL-33R was reduced compared with controls (Fig. 1e). We next assessed whether human ILC2s expressed RBM3 and if TSLP and IL-33 regulated RBM3 expression as they do in mouse ILCs. FACS purified human ILC2s stimulated with a combination of TSLP and IL-33 demonstrated significantly increased RBM3 immunostaining after 24 h and 72 h compared with control staining (Supplementary Fig. 4). Thus, activation of ILCs by epithelial cytokines IL-33 and TSLP led to increased RBM3 expression in both mice and humans, suggesting studies of RBM3 in mouse ILCs might be relevant to humans.

### RBM3 suppresses Alternaria-induced type 2 lung inflammation

We next performed in vivo asthma model experiments with *Rbm3*^*−/−*^ mice to assess lung inflammatory and ILC responses. We analyzed lineage-negative Thy1.2^+^ lung ILCs from naïve *Rbm3*^*−/−*^ mice and found no significant changes in the absolute or relative number of ILCs between naïve WT and *Rbm3*^*−/−*^ mice (Supplementary Fig. 5a, b). Naive *Rbm3*^*−/−*^ ILCs had similar surface marker expression of common ILC2 surface markers when compared to WT ILC2s (Supplementary Fig. 5c), and naïve *Rbm3*^*−/−*^ mice also showed similar levels of BAL and lung eosinophils as WT controls (Supplementary Fig. 5d, e). Thus, *Rbm3*^*−/−*^ mice are phenotypically similar to WT mice in terms of baseline lung eosinophil and ILC levels.

**Fig. 2:**
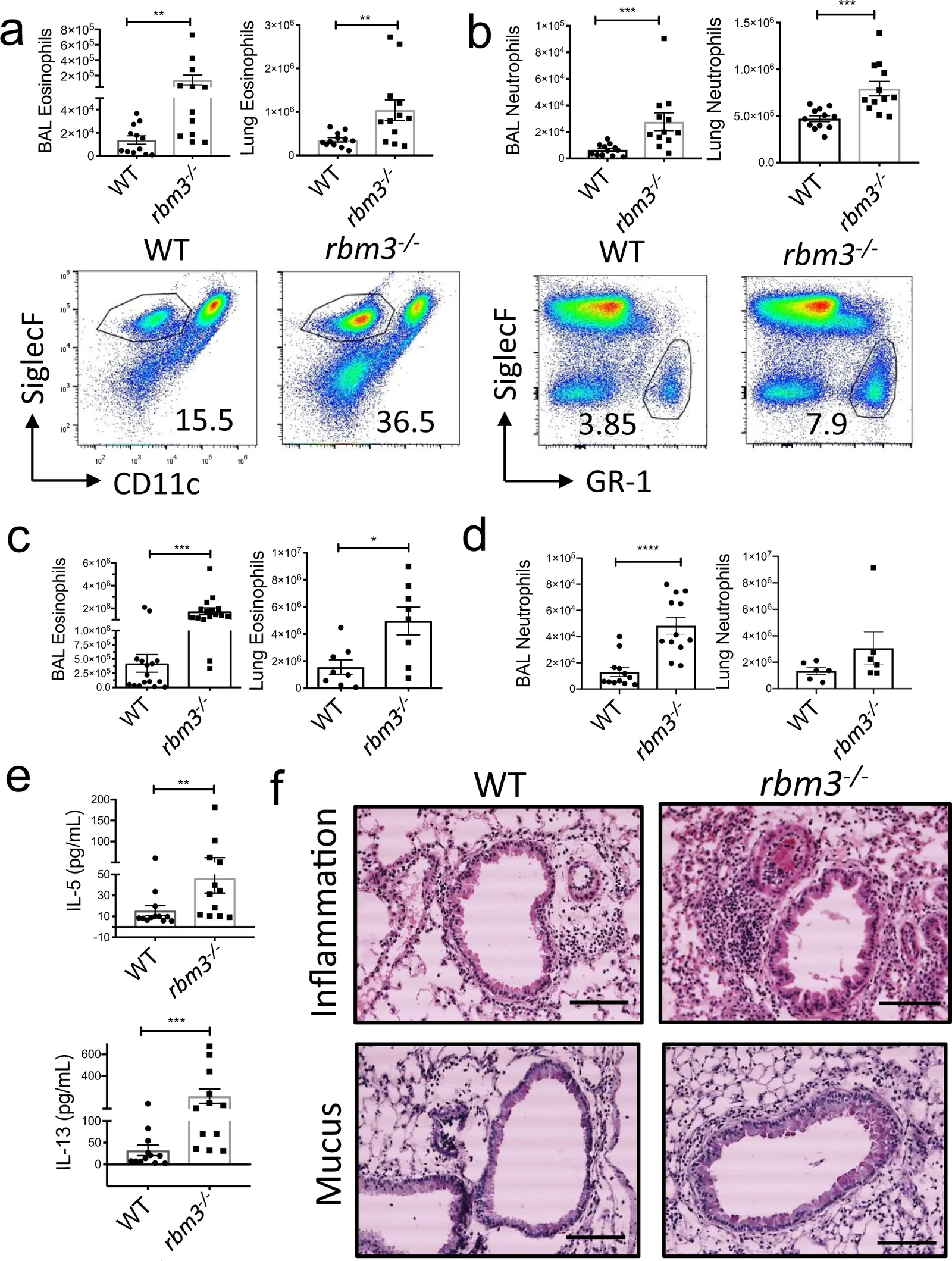
*Alternaria-*challenged *Rbm3*^*−/−*^ mice exhibit significant increases in airway granulocytes, inflammation, and Th2 cytokines. Wild-type and *Rbm3*^*−/−*^ mice were challenged with 10 µg *Alternaria* three times over 7 days. Data shown are representative of 3 experiments (4 mice per group). **a** Total BAL (*p* = 0.0036) and lung eosinophils (*p* = 0.0083). Representative FACS plots of WT and *Rbm3*^*−/−*^mice. Mann-Whitney Test, two-tailed. **b** Total BAL (p = 0.0001) and lung (*p* = 0.0001) neutrophils. Representative FACS plots of WT and *Rbm3*^*−/−*^ mice. Statistics analyzed by Mann-Whitney Test, two-tailed. Wild-type and *Rbm3*^*−/−*^ mice were challenged with 20 µg and 10 µg *Alternaria* over the span of 10 days. Data shown are representative of 4 experiments (4 mice per group). **c** Total BAL (*p* = 0.0001) and lung (*p* = 0.0148) eosinophils. Mann-Whitney Test, two-tailed. **d** Total BAL (*p* < 0.0001) and lung (*p* = 0.2087) neutrophils. Unpaired *t*-test, two-tailed. **e** BAL levels of Type 2 cytokines (IL-13 p = 0.0005 and IL-5 *p* = 0.0036). Mann-Whitney Test, two-tailed. **f** H&E and PAS lung sections at 20X; scale bar is 100 µm. Airway images representative of 4 mice per group. *p < 0.05, ** *p* < 0.01, ***p < 0.001, ****p < 0.0001. Data are presented as mean values+/*−* SEM.

To ascertain potential effects of RBM3 in vivo, we repetitively challenged WT and *Rbm3*^*−/−*^ mice with *Alternaria*, a fungal allergen that induces potent type 2 lung inflammation and ILC activation^21,26,27^. *Rbm3*^*−/−*^ mice challenged with *Alternaria* three times over 7 days developed significant increases in BAL and lung eosinophils (Siglec-F^+ ^^+^CD11c- cells) and neutrophils (Siglec-F^−^GR-1^+^ cells) compared to WT mice (Fig. 2a, b). Similarly, *Rbm3*^*−/−*^ mice receiving 4 challenges over 10 days also showed increased eosinophils and neutrophils in BAL and lung (Fig. 2c, d) as well as increased BAL levels of IL-5 and IL-13 (Fig. 2e). Further, lung sections stained for H&E and PAS displayed increased peribronchial inflammation and mucus production in *Rbm3*^*−/−*^ mice (Fig. 2f). Thus, *Rbm3*^*−/−*^ mice exhibited increased type 2 inflammation compared to WT mice in multiple *Alternaria* challenge models.

### RBM3 suppresses Alternaria-induced lung ILC responses

**Fig. 3:**
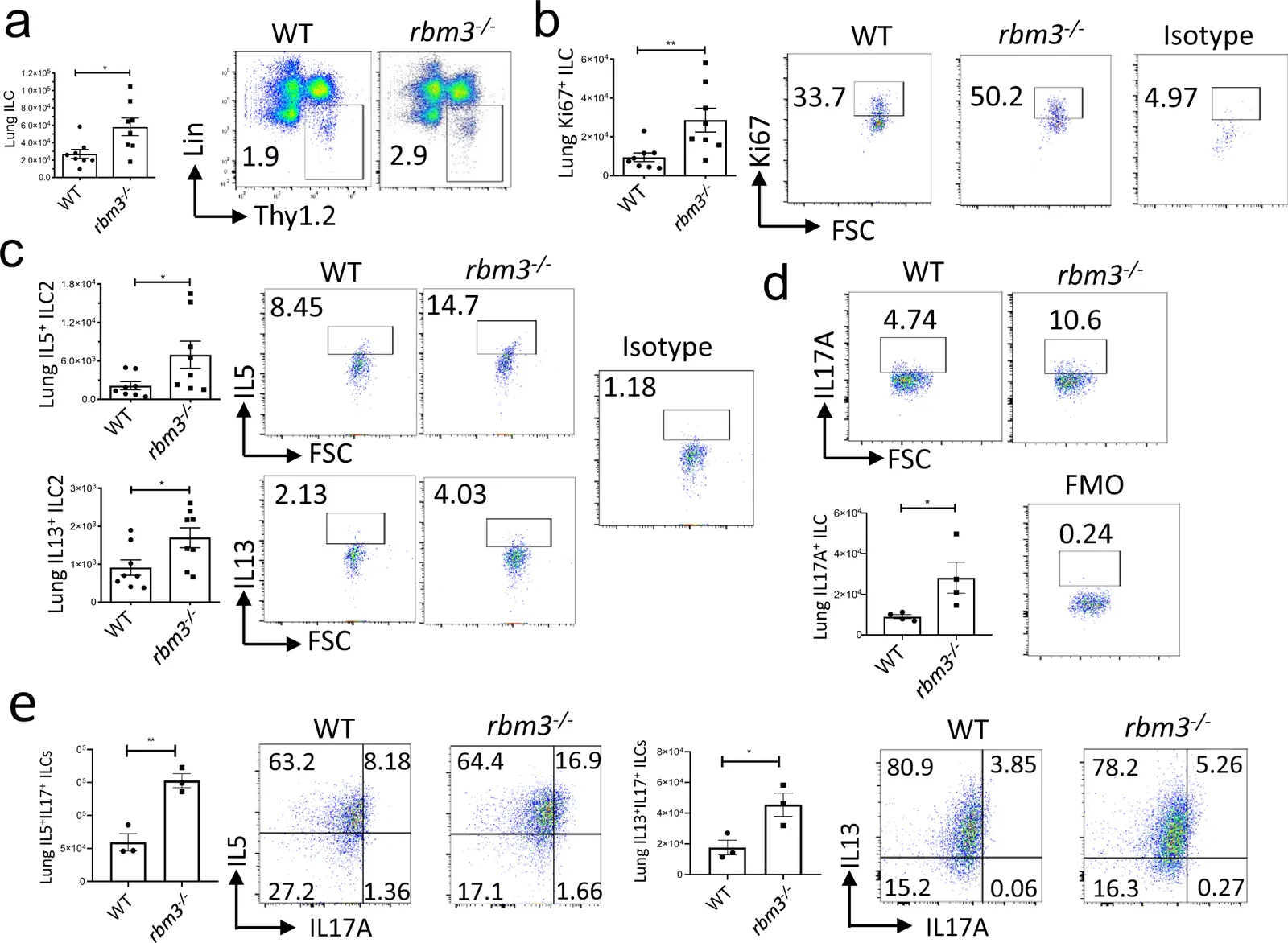
ILC2s are increased in *Rbm3*^*−/−*^ mice and show increased Th2 and IL-17 cytokine production. Wild-type and *Rbm3*^*−/−*^ mice were challenged with 20 µg and 10 µg *Alternaria* over the span of 10 days. Data shown are representative of 4 experiments (4 mice per group). **a** Lung Lin^-^T1ST2^+^ ILC totals (*p* = 0.0207) and FACS plots of Lin^-^Thy1.2^+^ population. Mann-Whitney Test, two-tailed. **b** Totals of Ki-67 expressing lung ILCs (*p* = 0.0070). FACS plots of Ki-67 percentages and an isotype control. Mann-Whitney Test, two-tailed. **c** Total IL5 (*p* = 0.0379) and IL13 (*p* = 0.0379) expressing lung ILCs. FACS plots of type 2 cytokines percentages and an isotype control. Mann-Whitney Test, two-tailed. Data representative of 4 mice per group. **d** Total IL17A-expressing ILCs (*p* = 0.0478) and representative FACS plots showing IL17A^+^ ILC percentages. Unpaired t-test, two-tailed. Data representative of 4 mice. WT and *Rbm3*^*−/−*^ mice were challenged intranasally with 25 μg *Alternaria* 3 times over 7 days. (**e**, top) Total number and percent of ILCs producing IL5 and IL17 in WT and *Rbm3*^*−/−*^ mice (*p* = 0.0053). FACS plots are representative of one experiment with 3 mice per group. (**e** bottom) Total number and percent of ILCs producing IL13 and IL17 in WT and *Rbm3*^*−/−*^ mice. (*p* = 0.0360). FACS plots are representative of one experiment with 3 mice per group, isotype controls shown for **b**–**e**. Unpaired *t*-Test, two-tailed. *p < 0.05, **p < 0.01, ***p < 0.001. Data are presented as mean values+/*−* SEM.

We next measured ILC activation and proliferation in the lungs of *Rbm3*^*−/−*^ mice challenged with *Alternaria*. Lung ILCs identified as CD45^+^ lineage-negative Thy1.2^+^ lymphocytes were increased in both number and percent in *Rbm3*^*−/−*^ mice compared with WT mice (Fig. 3a). Further, lung Ki-67^+^ proliferating ILCs were also increased significantly in *Rbm3*^*−/−*^ mice (Fig. 3b). ILC2s identified by their production of IL-5 and IL-13 were higher in the *Rbm3*^*−/−*^ mice compared with WT mice (Fig. 3c). Interestingly, ILCs from *Rbm3*^*−/−*^ mice demonstrated significantly higher production of IL-17A which may be produced by iILC2s (or ILC2-17s) and/or ILC3s (Fig. 3d)^2,9,10^. Dual staining of IL-5^+^IL-17^+^ ILCs and IL-13^+^IL-17a^+^ ILCs showed increases in IL-17 from Th2 cytokine-producing *Rbm3*^*−/−*^ ILCs compared to WT ILCs, suggesting that these cells may be ILC2_17_ cells (Fig. 3e, Supplementary Fig. 5f).

The effects of global RBM3 deficiency were not limited to ILCs. After *Alternaria*-challenges, the total number of ST2^+^ CD4^+^ T cells in BAL and lung were greater in *Rbm3*^*−/−*^ mice compared to WT control (Supplementary Fig. 6a). Ki-67 expression within ST2^+^ T cells was also significantly increased in *Rbm3*^*−/−*^ mice. ST2 expression within T cells also trended higher in *Rbm3*^*−/−*^ mice lung and BAL (Supplementary Fig. 6b, c). Thus, it is plausible that RBM3 also directly or indirectly suppresses CD4^+^ Th2 cell responses.

**Fig. 4:**
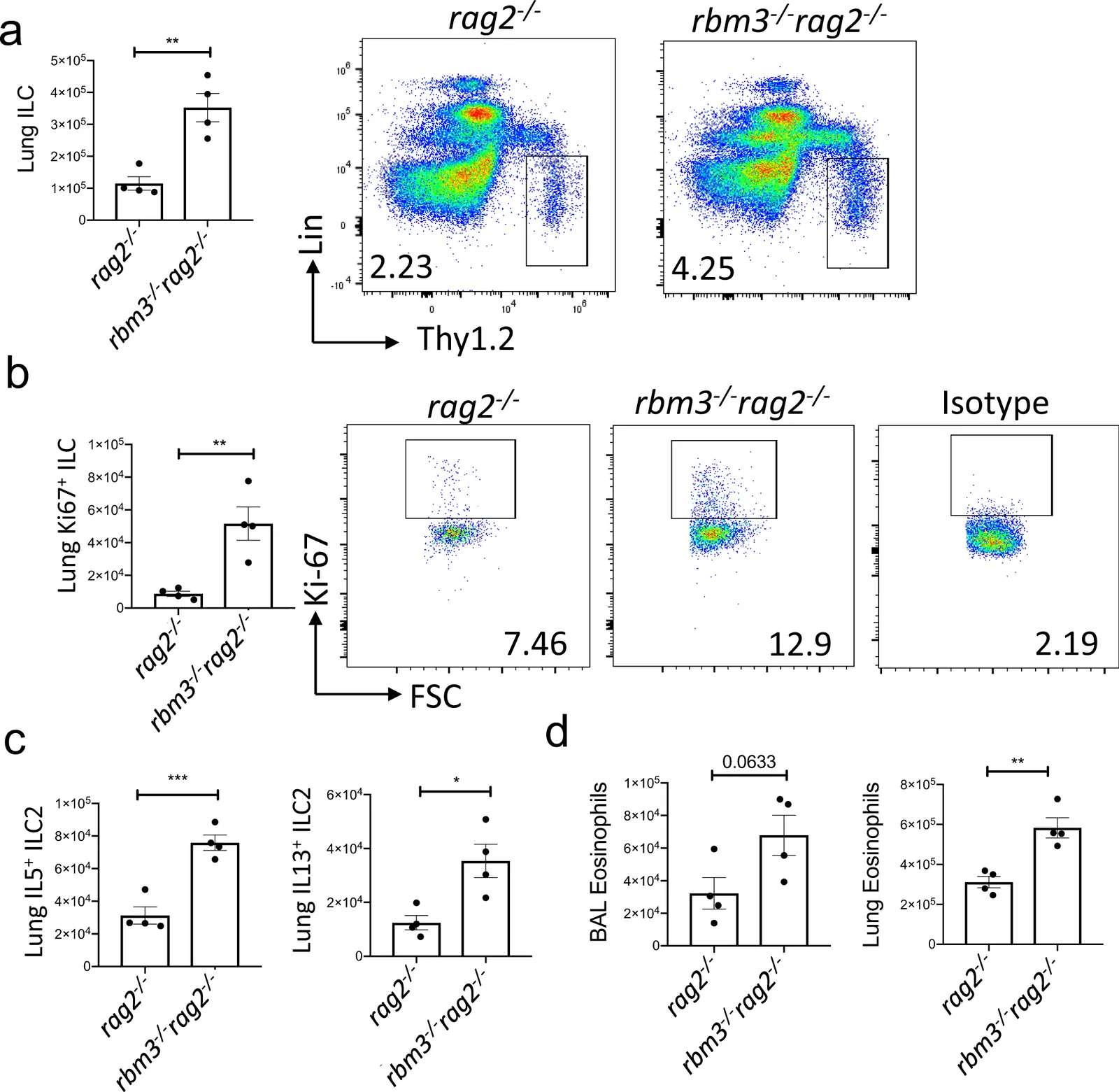
RBM3 suppresses lung ILC2s independent of adaptive immunity. *Rag2*^*−/−*^ and *Rbm3*^*−/−*^*Rag2*^*−/−*^ mice were challenged with 20 µg *Alternaria* four times over 10 days. Data representative of 4 mice per group. **a**–**c** Total number of ILCs (*p* = 0.0029), Ki67 expressing ILCs (*p* = 0.0060), and type 2 cytokine-producing ILC2s (IL-5 *p* = 0.0008 and IL-13 *p* = 0.0145). Unpaired t-Test, two-tailed. **d** Total BAL (*p* = 0.0633) and lung (*p* = 0.0034) eosinophils. Unpaired *t*-Test, two-tailed. *p < 0.05, **p < 0.01, ***p < 0.001. Data are presented as mean values+/*−* SEM.

To better assess the function of RBM3 in ILCs independent of T cells, we created double deficient *Rbm3*^*−/−*^*Rag2*^*−/−*^ mice. Consistent with the findings in single knockout *Rbm3*^*−/−*^ mice, the *Rbm3*^*−/−*^*Rag2*^*−/−*^ mice demonstrated significantly higher lung ILCs, Ki-67-expressing ILCs, and type 2 cytokine-producing ILC2s compared to *Rag2*^*−/−*^ mice (Fig. 4a–c). BAL and lung eosinophils were also significantly increased in the double knock-out mice compared to controls (Fig. 4d). Overall, these results show a suppressive effect of RBM3 in type 2 lung inflammation and ILC activation, even in the absence of adaptive immunity.

### RBM3 directly suppresses IL-33-induced ILC responses in vitro and in vivo

**Fig. 5:**
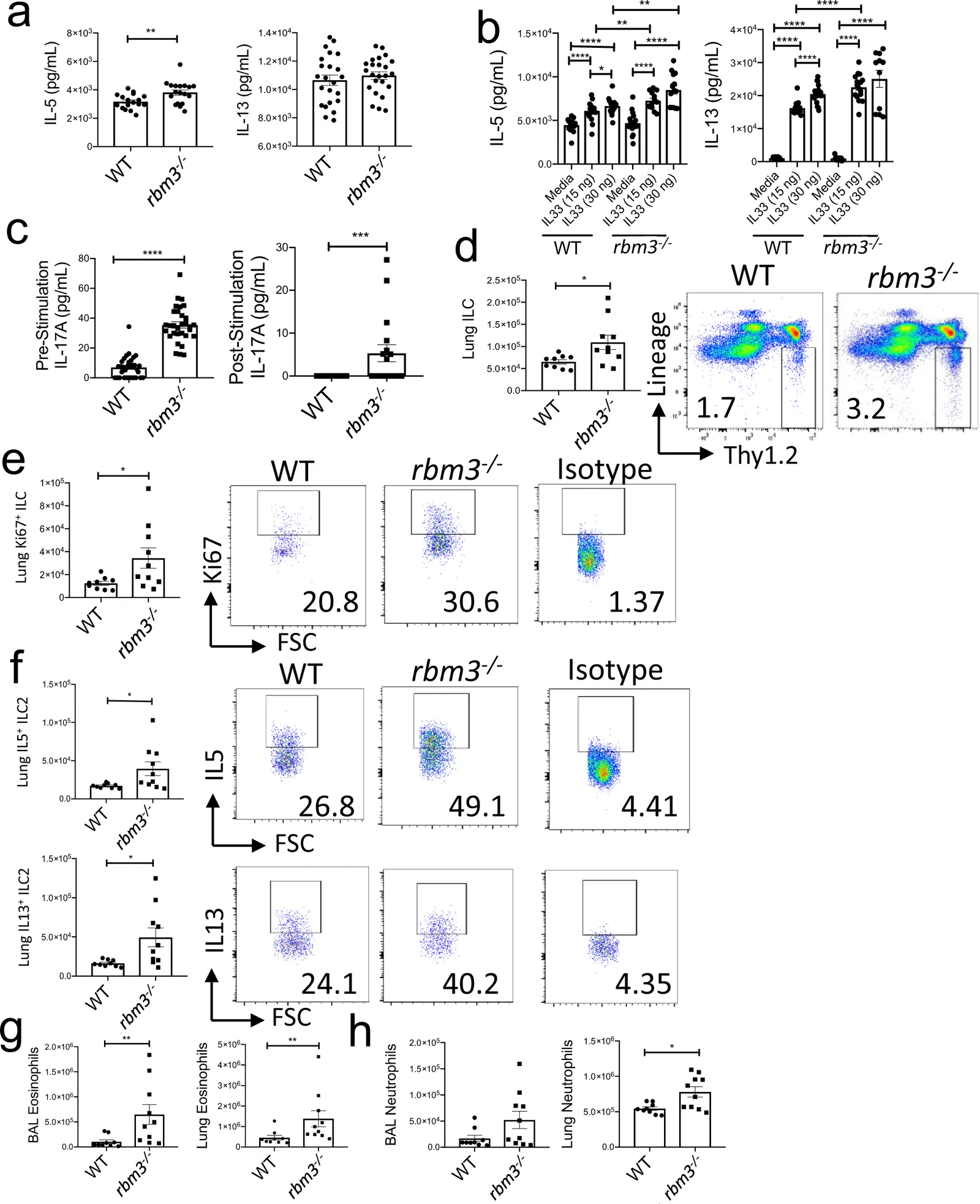
RBM3 shows a suppressive effect in ILC2s stimulated with IL-33 in vitro and in vivo. WT and *Rbm3*^*−/−*^mice were intranasally challenged four times with 50 µg *Alternaria* over 10 days. Lin^-^Thy1.2^+^ ILC2s were FACS sorted from mouse lung and were rested with 10 ng/mL IL-2 and IL-7 for 48 h prior to stimulation with IL-33. (A) IL-5 (*p* = 0.0073) and IL-13 (*p* = 0.4316) concentration pre-stimulation. Mann-Whitney Test, two-tailed. *n* = 17–24 samples from 2 experiments. **b** IL-5 and IL-13 concentration post-stimulation with 15 ng and 30 ng IL-33. Mann-Whitney Test, two-tailed. *n* = 13–20 samples from 2 experiments. *p < 0.05, **p < 0.01, ***p < 0.001, ****p < 0.0001 **c** IL-17A concentration levels pre-stimulation (*p* < 0.0001; *n* = 31–32 samples) and post-stimulation (*p* = 0.0006; *n* = 17 and 20 samples) with 30 ng IL-33. Mann-Whitney Test, two-tailed. WT and *Rbm3*^*−/−*^ mice were intranasally challenged with 10 ng IL-33 three times over 7 days. *n* = 9–10 mice per group. **d** Total Lin^-^Thy1.2^+^ ILCs (*p* = 0.0172) and representative FACS plots^.^ Mann-Whitney Test, two-tailed. **e** Total Ki-67-expressing ILCs (*p* = 0.0279) and representative FACS plots, isotype shown. Mann-Whitney Test, two-tailed. **f** Total number of IL5-expressing ILC2s (*p* = 0.0220) and IL13-expressing ILC2s (*p* = 0.0101) and representative FACS plots of cytokine levels. Cells were cultured for 3 h with cell stimulation cocktail prior to staining, isotypes shown in E&F. Mann-Whitney Test, two-tailed. **g** Total BAL (*p* = 0.0041) and lung (*p* = 0.0057) eosinophils. Mann-Whitney Test, two-tailed. **h** Total BAL (*p* = 0.1564) and lung (*p* = 0.0279) neutrophils. Mann-Whitney Test, two-tailed. *p < 0.05, **p < 0.01, ***p < 0.001, ****p < 0.0001. Data are presented as mean values+/*−* SEM.

To establish whether RBM3 directly regulated ILC responses, we performed studies with IL-33-stimulated ILCs in vitro and in vivo. FACS sorted WT and *Rbm3*^*−/−*^ lung Lin^−^Thy1.2^+^ ILCs from *Alternaria-*challenged mice were rested for 48 h in media alone prior to stimulation. Immediately prior to stimulation, *Rbm3*^*−/−*^ ILC2s produced significantly more IL-5 by ELISA than wild-type ILC2s (Fig. 5a). In contrast, there were no differences in the production of IL-13 prior to stimulation. IL-33 stimulation (15 ng/ml) of *Rbm3*^*−/−*^ ILCs resulted in increased IL-5 and IL-13 compared with wild-type ILCs (Fig. 5b). ILCs lacking RBM3 produced significantly more IL-5 after stimulation with IL-33 (both 15 and 30 ng/ml IL-33). Consistent with data in Fig. 3d, ILC IL-17A production was increased in *Rbm3*^*−/−*^ ILCs after 48 h of rest (Fig. 5c). Unlike the Type 2 cytokines, IL-17A was not further increased above pre-stimulation conditions with IL-33 stimulation (Fig. 5c). These results demonstrate a direct inhibitory regulatory function of RBM3 in ILC type 2 cytokine production in response to IL-33, as well as a suppressive effect of RBM3 in IL-17A production measured ex vivo.

To assess a direct effect of RBM3 function in ILCs in vivo, *Rbm3*^*−/−*^ and WT mice were administered IL-33 intranasally which directly activates lung ILC2s^27,28^. Similar to *Alternaria*-challenged mice, direct challenge with IL-33 led to significantly greater lung Lin^−^Thy1.2^+^ ILCs as well as Ki-67^+^ proliferating ILCs in *Rbm3*^*−/−*^ mice compared to WT control mice (Fig. 5d, e). Further, *Rbm3*^*−/−*^ lung cells had significantly higher IL-5 and IL-13 producing ILC2s (Fig. 5f). BAL and lung eosinophilia were also significantly increased in *Rbm3*^*−/−*^ mice (Fig. 5g). Overall, neutrophils in BAL and lung were also increased in *Rbm3*^*−/−*^ mice treated with IL-33 (Fig. 5h). Thus, RBM3 directly suppresses lung ILC activation by IL-33 in vitro and in vivo, and this leads to significant differences in eosinophilia and neutrophilia in vivo.

### RBM3 intrinsically suppresses lung ILC2 responses

**Fig. 6:**
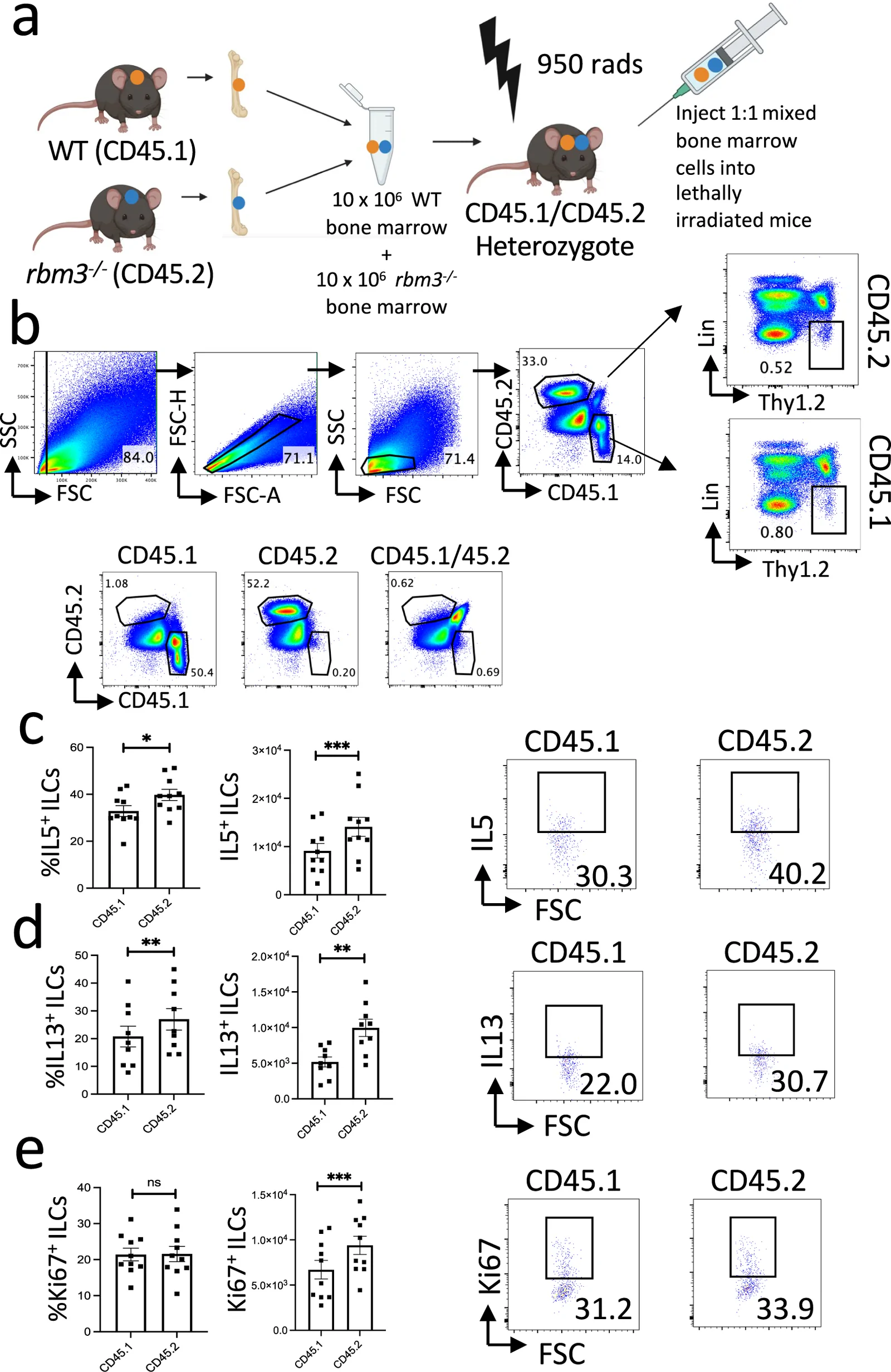
Mixed bone marrow chimeras show cell-intrinsic exaggeration of a type 2 response in ILCs from *Rbm3*^*−/−*^ mice. **a** Schematic of bone marrow reconstitution. Mixed bone marrow chimeras were generated using bone marrow from CD45.1 *Rbm3*^+/+^ (WT) mice and CD45.2 *Rbm3*^*−/−*^ mice and reconstituted for 10–12 weeks, images created with BioRender.com. **b** Mixed bone marrow chimera mice were challenged 3 times over 7 days with 20 ${{{{\rm{\mu }}}}}$g *Alternaria*. FACS plots are representative of 2 independent experiments (5 mice each, *n* = 10) with biological controls. ILCs are gated as Lineage^−^ Thy1^.^2^+^ cells. **c** Percent (*p* = 0.0178) and total (*p* = 0.0003) IL5 expressing ILCs from WT vs. *Rbm3*^*−/−*^mice. FACS plots of IL5 percentages. **d** Percent (*p* = 0.0012) and total IL13 (*p* = 0.0029) expressing ILCs from WT vs. *Rbm3*^*−/−*^mice. Representative FACS plots of IL13 percentages. **e** Percent (*p* = 0.7764) and total Ki67 (*p* = 0.0001) expressing ILCs from WT vs *Rbm3*^*−/−*^mice. Representative FACS plots of Ki67 percentages. Paired *t*-Test, two-tailed. *p < 0.05, **p < 0.005, ***p < 0.0005.

We next generated mixed bone marrow chimera mice by irradiating congenic heterozygote mice (CD45.1^+^CD45.2^+^) and injecting a 1:1 ratio (10 × 10^6^ cells each) of WT and *Rbm3*^*−/−*^ bone marrow cells (Fig. 6a). After 10–12 weeks of reconstitution, mice were challenged with 20 μg *Alternaria* three times over 7 days. ILC Ki-67 expression and type 2 cytokine production was analyzed from Lin^−^Thy1.2^+^ ILCs from CD45.1^+^ or CD45.2^+^ bone marrow cells (Fig. 6b, Supplementary Fig. 8c). Type 2 cytokine production from CD45.2^+^ *Rbm3*^*−/−*^ ILCs was significantly increased compared to WT ILCs, as shown in the percentage and absolute number of IL-5^+^ ILC2s (Fig. 6c, Supplementary Fig. 8c) as well as the percentage and absolute number of IL-13^+^ ILC2s (Fig. 6d, Supplementary Fig. 8c). In addition, Ki-67 expression was significantly increased in *Rbm3*^*−/−*^ ILCs compared to WT ILCs (Fig. 6e). In contrast, *Rbm3*^*−/−*^ CD45.2^+^Lin^+^ Thy1.2^+^ cells, which are largely made up of T cells, did not show any change in proliferation or Type 2 cytokine production (Supplementary Figs. 7, 8d) suggesting a more selective intrinsic effect on ILCs. CD45.2^+^ *Rbm3*^*−/−*^ lung BAL eosinophils were only slightly increased over CD45.1^+^ WT eosinophils (Supplementary Fig. 8a, b).

### Activated ILC programs including increased CysLT1R expression are present in the *Rbm3*^*−/−*^ ILC transcriptome

**Fig. 7:**
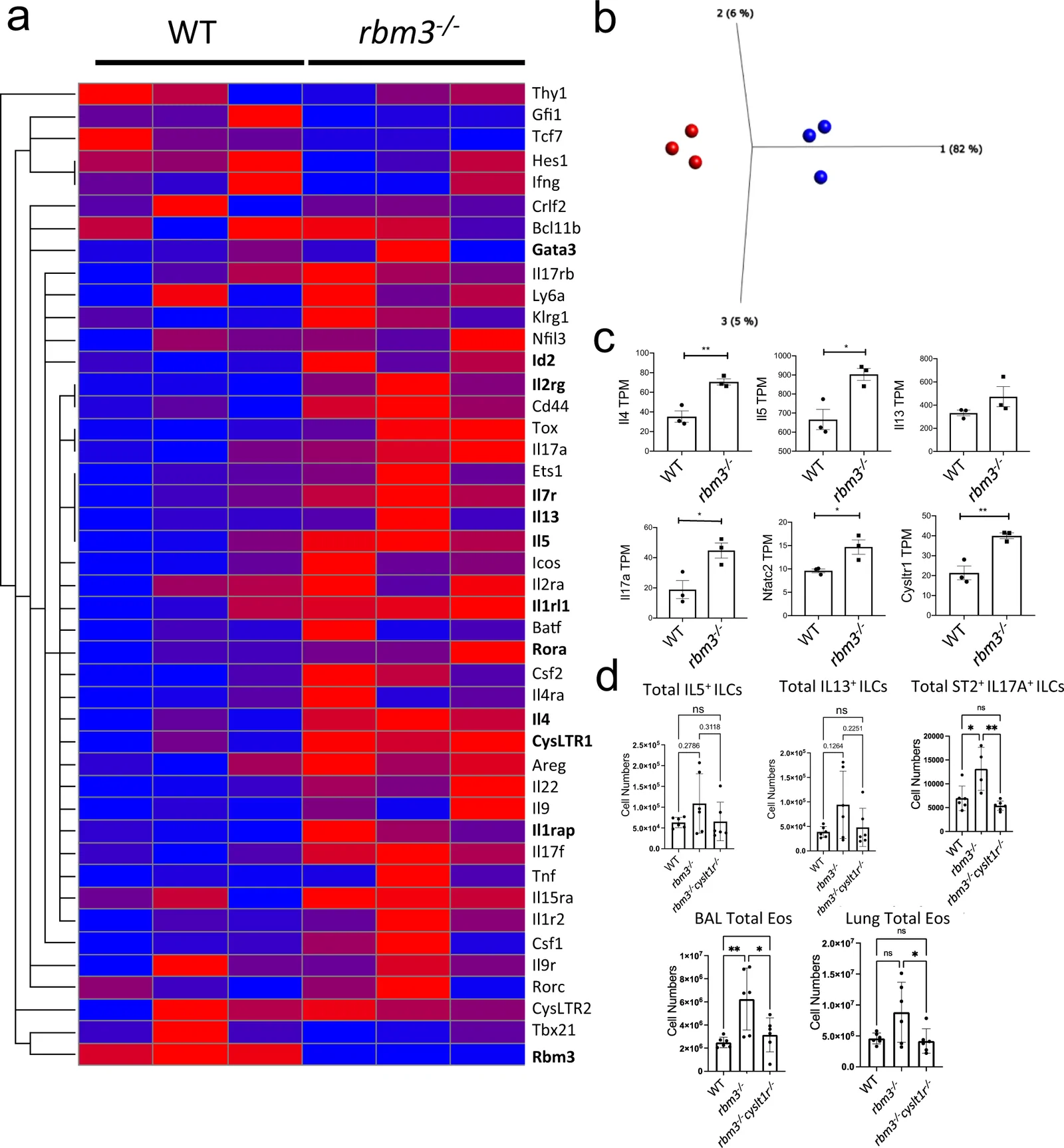
CysLT1R controls eosinophilia and ST2^+^IL-17^+^ ILC2 accumulation in *Rbm3*^*−/−*^ mice. WT and *Rbm3-/-* mice were challenged with 25 µg *Alternaria* three times over 7 days. Lin^-^Thy1.2^+^ ILCs were FACS-sorted and bulk RNA-sequenced. Data representative of three mice per group. **a** Relative transcript levels of select ILC transcripts **b** PCA plot of WT (blue) and *Rbm3*^*−/−*^ (red) samples. **c** TPMs of select surface marker, cytokine, and transcription factor transcripts. Unpaired t-test, two-tailed. *p < 0.05, **p < 0.01, ***p < 0.001. **d** Wild-type, *Rbm3*^*−/−*^, and *Rbm3*^*−/−*^*Cyslt1r*^*−/−*^ mice were challenged with *Alternaria* three times over 14 days. Total type 2 cytokine-producing ILC2s, IL17 producing ST2^+^ILCs, and BAL and lung eosinophils in WT, *Rbm3*^*−/−*^, and *Rbm3*^*−/−*^*Cysltr1*^*−/−*^ mice, One^*-*^way ANOVA. *p < 0.05, **p < 0.01, ***p < 0.001. Data are presented as mean values+/*−* SEM.

We next performed RNA-seq analysis of FACS-sorted Lin^−^Thy1.2^+^ ILCs from WT and *Rbm3*^*−/−*^ mice (99.4% pure) to more broadly understand the mechanisms by which RBM3 inhibits lung ILC responses (Supplementary Fig. 9). Heat map analysis of common ILC transcripts, particularly those related to ILC2s and ILC3s, were more highly expressed in *Rbm3*^*−/−*^ ILCs than WT controls (Fig. 7a). Principle component analysis showed clear consistent global differences between WT and *Rbm3*^*−/−*^ ILC transcriptomes (Fig. 7b). Several candidate cytokine transcripts were greater in *Rbm3*^*−/−*^ ILCs compared with WT ILCs including *Il4*, *Il5*, *Il13*, *Areg*, *Tnf*, *Il17a*, *Il17f*, and *Il22* (Fig. 7c, Supplementary Fig. 10a). The ILC1 cytokine *Ifng* was not significantly different in *Rbm3*^*−/−*^ ILCs (Supplementary Fig. 10a) and may reflect that RBM3 modulates type 2 cytokine responses and IL-17a production from ILCs or an overall lack of ILC1 responses in the *Alternaria* model. *Rbm3*^*−/−*^ ILCs also demonstrated increases in relevant ILC surface markers including *Il1rl1, Il2rg*, *Cysltr1*, *Il1rap*, *Cd44, Klrg1*, and *Icos* (Supplementary Fig. 10b). Interestingly, while type 2 cytokine transcripts were upregulated in *Rbm3*^*−/−*^ ILCs, *Gata3* levels were not increased at a transcript or protein level (Supplementary Fig. 10c, g) suggesting other signaling factors are responsible for the increased ILC type 2 cytokine production in *Rbm3*^*−/−*^ ILCs. Transcription factors known to influence the development, differentiation, or activation of ILCs (including *Tox*^29^, *Ets*1^30^, *Rora*^31,32^, *Irf4*^33^, and *Ed2*^1^) were upregulated in *Rbm3*^*−/−*^ ILCs, (Supplementary Fig. 10c). Anti-apoptotic and survival transcripts, including *Bcl2*^1^, *Cflar*^34^, *Bcl2a1d*^35^, and *Hbixip*^36,37^, were also moderately or significantly upregulated in *Rbm3*^*−/−*^ ILCs (Supplementary Fig. 10d). BCL2 protein expression was also higher in ILCs from *Alternaria*-challenged *Rbm3*^*−/−*^ mice than WT controls (Supplementary Fig. 10e) suggesting a potential survival benefit to the *Rbm3*^*−/−*^ ILCs. Id2 protein expression was also higher in ILC from *Alternaria*-challenged *Rbm3*^*−/−*^ mice (Supplementary Fig. 10f). *Rbm3*^*−/−*^ ILCs had increased *Nfactc2* expression (encodes NFAT1) which could be a candidate mechanism responsible for increased ILC type 2 cytokine production as NFAT1 mediates ILC2 activation via CysLTR1^10,38,39^ to produce Th2 and Th17 cytokines (Fig. 7c). Though RBM3 binds to the AU-rich regions of mRNAs, there was no correlation between the number of AUUUA (preferentially bound by RBM3) or total ARE regions and the log fold change of differentially expressed transcripts (Supplementary Fig. 11). Therefore, the AU-rich density of transcripts was not predictive of RBM3 regulation of ILC transcriptome differences.

### Enhanced lung inflammation and ST2^+^IL-17^+^ ILC accumulation in *Rbm3*^*−/−*^ mice is dependent on CysLT1R

Given the increased expression of CysLT1R and NFAT1 in *Rbm3*^*−/−*^ ILCs, we crossed *Rbm3*^−^^/^^−^ mice to *Cyslt1r*^*−/−*^ mice to generate *Rbm3*^*−/−*^*Cyslt1r*^*−/−*^ double knockout mice that were then challenged with *Alternaria* three times for 14 days. As expected, there was an increase in lung ILC type 2 cytokine and IL-17a production and eosinophilia in *Rbm3*^*−/−*^ mice compared to WT mice (Fig. 7d). ILC IL-5 and IL-13 production trended toward reduction, but not significantly, in *Rbm3*^*−/−*^*Cyslt1r*^*−/−*^ mice compared to *Rbm3*^*−/−*^ mice. However, ST2^+^IL-17^+^ ILCs (ILC2-17s) as well as eosinophils were significantly reduced in double knockout mice. Taken together, this suggests that CysLT1R contributes to RBM3-mediated suppression of lung eosinophilia and ILC2-17 responses.

## Discussion

Innate lymphoid cells including ILC2s have recently emerged as critical contributors to immune-mediated diseases including asthma^40,41^. The majority of the ILC literature thus far has reported soluble factors that activate or inhibit ILC2 function including cytokines and lipid mediators^42^. However, an understanding of intracellular mechanisms that more broadly control ILC function might provide important insights into ILC-driven immune diseases. Studies thus far have demonstrated that specific miRNAs activate ILC2s through multiple mechanisms^15,16^. Here, we found that RBM3 is a highly expressed RNA-binding protein (RBP) in lung ILC subsets which is induced by epithelial cytokines IL-33 and TSLP, and negatively regulates ILC type 2 and 17 cytokine production. Mixed bone marrow congenic chimera mice displayed increased type 2 cytokine production in ILCs from *Rbm3*^*−/−*^ mice, highlighting an ILC-intrinsic activity of RBM3. Further, transcriptomic analysis demonstrated broad differences in *Rbm3*^*−/−*^ ILCs including expression of CysLT1R, which we found to regulate ILC2 IL-17^+^ cell and eosinophil accumulation in *Rbm3*^*−/−*^ mice.

RBM3 is a 17KD RNA-binding protein that promotes mRNA stability and translation efficiency by binding to ARE binding regions. Very little is understood about RBM3 in inflammation and immunity. One report showed that RBM3 is downregulated in febrile illness and knockdown of RBM3 led to increases in miRNAs that suppress *PGE2*, *IL6*, and *IFNA1*^23^. However, earlier studies showed that RBM3 deficient mice had normal numbers of NK, T, and B cells and had no differences in innate cytokine responses to the TLR9 ligand CpG^43^. Stressors such as tissue hypoxia and low temperatures can induce RBM3 expression^44^. Our work demonstrates that RBM3 is induced by IL-33 and/or TSLP in mouse and human ILC2s. Of note, IL-33 has been reported to promote a tumor hypoxic microenvironment, with generation of reactive oxygen species, that could lend toward an indirect induction of RBM3 through local hypoxia^45,46^. Further, in addition to hypoxia and hypothermia, activation of NF-κB promotes RBM3 expression and extracellular IL-33 activates NF-κB^47,48^. Thus, there may be multiple mechanisms by which IL-33 and/or TSLP may induce RBM3 expression under inflammatory stress conditions.

We found that RBM3 suppresses the classic ILC2 cytokines (IL-5 and IL-13) as well as IL-17A and ILC proliferation. Furthermore, exacerbation of type 2 responses in *Rbm3*^*−/−*^ mice was independent of changes in total ILC GATA3 levels and lacked correlation with number of ILC AU-rich element (ARE) transcripts. RBM3 has a multitude of complex potential mechanisms that regulate cellular changes during stress including controlling translation efficiency through ARE binding, protein-protein interactions, and effects on miRNAs directly or through dicer processing^17,23,44^. Interestingly, RBM3 has also been reported to inhibit the p38 MAP kinase pathway which promotes cytokine production by ILC2s in response to IL-33^49–51^. Thus, removal of RBM3’s inhibition of the p38 pathway in ILCs may result in increased cytokine production in *Rbm3*^*−/−*^ ILCs. Our studies also demonstrate that RBM3 has no effect on ILC2 numbers and lung eosinophilia under homeostatic naïve conditions but has a clear suppressive function during type 2 inflammatory insult. This is consistent with RBM3 being previously identified as a “stress-response” protein that is largely protective in neural survival in conditions of stress^44^. Our data suggest that RBM3 limits hyperactive ILC responses which could be an important protective mechanism during lung inflammation.

In this study, we took a broad approach to ILC identification and included lineage-negative Thy1.2^+^ lymphocytes as ILCs based on our recent work showing that CD127 and ST2 exclude approximately 40% of Th2 cytokine-producing ILC2s^21^. Further, several studies have demonstrated significant heterogeneity and plasticity of ILCs (reviewed in^52^). For example, in addition to conventional ILC3s, IL-17 production occurs from other ILC sources including “inflammatory” iILC2s (or ILC2-17s) induced by IL-25, cysteinyl leukotrienes, and Notch signaling^9,10,53^. Initial reports showed that ST2-negative ILC2s were “inflammatory” ILCs that produce IL-17A in response to IL-25^9^. However, Cai et al. subsequently reported that ST2^+^ ILC2s are also key IL-17A producers (known as ILC2-17s) which can also be induced by CysLTs^10^. Similarly, dual cytokine staining in our studies showed that ILC2s were the dominant ILC source of IL-17 in our models. Despite these results, we cannot exclude the presence of independent populations of ILC3s or other IL-17A-producing ILCs that are regulated by *Rbm3*^*−/−*^ mice in different contexts.

RBM3 is expressed in multiple immune cells, including eosinophils, macrophages, and T cells. However, we investigated the direct function of RBM3 in lung ILCs given the emerging functions of ILCs during lung inflammation. In the mixed bone marrow chimera studies, we show that Lin^−^Thy1.2^+^ *Rbm3*^*−/−*^ ILCs were more activated compared with congenic WT ILCs. In contrast, Lin^+^Thy1.2^+^ populations, which largely include T cells, did not demonstrate a significant change in proliferation of type 2 cytokine production between WT and *Rbm3*^*−/−*^ cells. Thus, it appears that there may be a more selective intrinsic contribution of RBM3 in ILCs during type 2 inflammation compared with T cells. However, Th2 cells were increased in the complete *Rbm3-/-* mice after *Alternaria* challenges and this may be due to early ILC2 contributions to adaptive Th2 responses as previously reported^54,55^.

To explore potential mechanisms by which RBM3 regulates ILC biology, we performed transcriptomic studies which demonstrated global changes in activation of ILCs by RBM3 including differential expression of known ILC cytokines, receptors as well as transcription factor, and survival transcripts. Importantly, cytokine transcript data from *Rbm3*^*−/−*^ ILCs supported our in vivo findings that RBM3 suppressed type 2 and IL-17A cytokine production from ILCs. Transcripts for multiple receptors were increased in *Rbm3*^*−/−*^ ILCs, including *Il1rl1* (encodes ST2), *Il7r*, *Il2rg*, *Cysltr1*, and *Cd44*. Though *Il1rl1* was increased at a transcript level, we did not detect increased ST2 at a protein level by flow cytometry. Notably, several ILC transcription factors were increased in *Rbm3*^*−/−*^ ILCs including *Tox*, *Ets1*, *Rora*, *Irf4*, and *Id2*^1,29–33^. RORα and ETS1 also promote ILC2 cytokine production suggesting ILC2 cytokine production could be regulated by RBM3 through control of these transcription factors^30,56^. Despite differences in levels of developmental transcription factors in *Rbm3*^*−/−*^ ILCs, we did not detect differences in lung ILCs in naive *Rbm3*^*−/−*^ mice. This may be explained by induction of RBM3 in inflammatory settings which exerts effects on mature ILCs while being dispensable for ILC development.

Increased expression of the *Cysltr1* transcript in *Rbm3*^*−/−*^ ILCs was a particularly interesting finding because it was accompanied by an increase in expression of *Nfat2c* (encodes NFAT1). Previous reports have shown that CysLT1R signaling in ILCs is regulated by NFAT1 to promote increased Th2 and Th17 cytokine production^10,38,57,58^. Therefore, we generated *Rbm3*^*−/−*^*Cyslt1r*^*−/−*^ double knockout mice, which showed that CysLT1R regulates some of the hyperinflammatory phenotype observed in *Rbm3*^*−/−*^ mice after *Alternaria* challenges. Interestingly, ST2^+^IL-17^+^ ILCs were also controlled by CysLT1R in *Rbm3*^*−/−*^ mice which is in line with previous data that CysLTs induce IL-17 from ST2^+^ ILCs^10^. As ILC2 responses through CysLT1R are regulated by NFAT1, RBM3 suppression of the CysLTR1/NFAT1 axis could potentially limit ILC-driven lung inflammation though it is likely that other RBM3-dependent mechanisms contribute as well.

In summary, this work identifies an important function of RBM3, a highly expressed RNA-binding protein, in activated lung ILCs that is induced by IL-33 and TSLP. RBM3 dampens both type 2 cytokine and IL-17A cytokine production by lung ILCs and decreases downstream granulocyte infiltration in the setting of fungal allergen and IL-33 exposure. Mixed bone marrow chimera studies highlighted the cell-intrinsic effect of RBM3 on ILC function and transcriptomic analysis demonstrated RBM3 regulation of multiple cytokines, transcription factors, survival genes, and receptors critical to ILC function including CysLT1R. Studies of allergen-challenged *Rbm3*^*−/−*^*Cyslt1r*^*−/−*^ double knockout mice demonstrated that CysLTR1 contributes to RBM3-mediated ILC responses. The work presented herein further substantiates RNA-binding proteins as critical post-transcriptional regulatory mediators of ILC activation during type 2 inflammation.

## Methods

The research complies with all relevant ethical regulations and was approved by the UC San Diego IACUC animal care committee. Reagents for flow cytometry, qPCR, ELISA, allergen, and cytokines detailed in the Supplementary Data file.

### Mice

6–12-week-old female and male C57BL/6J mice were obtained from Jackson Laboratories (Bar Harbor, ME). Wild-type mice were age and gender matched to *Rbm3*^*−/−*^ mice acquired from Peter Vanderklish at TSRI and originally from Tadatsugu Taniguchi^43^ at University of Tokyo and bred in house. *Tslpr*^*−/−*^ mice were acquired from Dr. Michael Croft at the La Jolla Institute for Immunology and originally from Dr. Steven Ziegler^59^. The *Rbm3*^*−/−*^*Rag2*^*−/−*^ mice were created through multiple crosses of *Rbm3*^*−/−*^and *Rag2*^*−/−*^ mice and bred in house. The *Rbm3*^*−/−*^*Cyslt1r*^*−/−*^ mice were created through seven crosses of *Rbm3*^*−/−*^ and *Cyslt1r*^*−/−*^ mice (Jax stock number #030814) and bred in house. Mixed bone marrow chimera studies utilized CD45.1^+^ PEP boy homozygotes and CD45.1^+^CD45.2^+^ PEP boy heterozygotes (Jax stock number: #002014). WT and *Rag2*^*−/−*^ (Jax stock number #008449) mice originated from Jackson labs (Bar Harbor, ME). All mice were on a C57BL/6 background and controls were age and gender matched. All studies were approved by the University of California, San Diego Institutional Animal Care and Use Committee.

### Lung inflammation models

Mice were intranasally challenged with *Alternaria alternata* extract (Greer, Lenoir, NC) or recombinant IL-33 (R&D Systems, Minneapolis, MN) diluted in PBS. For the isolation of the ILC subsets for RNA-seq analysis and the isolation of Lin^−^Thy1.2^+^ ILCs for in vitro assays, mice were challenged with 50 µg *Alternaria* 4 times over 10 days. Mice were challenged with 25 µg *Alternaria* 3 times over 7 days to expand the Lin^−^Thy1.2^+^ ILC population for RNA-seq analysis. Experiments with *Tslpr*^*−/−*^ mice or the anti-IL-33R blocking antibody involved 4 challenges with *Alternaria*. Various *Alternaria* challenged models were utilized with the *Rbm3*^*−/−*^ mice. Mice were intranasally challenged three times over 7 days with 10 µg or 25 µg *Alternaria* or were challenged once with 20 µg *Alternaria* followed by 3 challenges of 10 µg *Alternaria* over 10 days where indicated. WT and *Rbm3*^*−/−*^ mice were also intranasally challenged with 10 ng recombinant IL-33 3 times over 7 days. *Rag2*^*−/−*^ and *Rbm3*^*−/−*^*Rag2*^*−/−*^ mice were challenged with 20 µg *Alternaria* 4 times over 10 days. Wild-type mice were treated with anti-IL-33 antibody (DJ8)^26^ or control IgG intraperitoneally on Days −1, 0, 3, and 6. For mixed bone marrow chimera studies, mice were challenged 3 times over 7 days with 20ug *Alternaria*. WT, *Rbm3*^*−/−*^, and *Rbm3*^*−/−*^*Cyslt1r*^*−/−*^ mice were challenged with 30ug *Alternaria* on D0 and 3ug *Alternaria* on D9 and D12 before being sacrificed on D14.

### Generation of mixed bone marrow chimeras

Bone marrow was isolated from femurs of donor CD45.2^+^ *Rbm3*^*−/−*^ mice and CD45.1^+^ WT congenic mice. Recipient CD45.1/CD45.2^+^ mice were lethally irradiated with 950 rads from a cesium irradiator. Irradiated mice were then injected retro-orbitally with a 1:1 ratio of 10×10^6^ CD45.1^+^ and 10×10^6^ CD45.2^+^ bone marrow cells. Mice were analyzed 8 weeks after injection by flow cytometry (single congenic CD45.1 and CD45.2 populations) to confirm successful bone marrow reconstitution.

### BAL and lung processing

Bronchoalveolar lavage (BAL) was collected in 2% BSA (Sigma, St. Louis, MO) and supernatant for the first flush of BAL was saved and stored in −20C for ELISA^26^. Lungs were collected in RPMI and was dissociated into a single-cell suspension using the Miltenyi Lung Digest Kit and Dissociator (Miltenyi Biotec, Bergisch Gladbach, Germany) per the company’s protocol. Cells counts were obtained using flow cytometry (Novocyte).

### Histology

The left half of the lung was used for histology. Lungs were perfused and fixed in 4% paraformaldehyde. Hematoxylin and Eosin (H&E) and Periodic acid–Schiff (PAS) staining was performed at the Histology Core in UCSD’s Moore’s Cancer Center and imaged with microscopy as previously reported^21^. Hematoxylin and Eosin (H&E) and Periodic acid–Schiff (PAS) staining were performed at the Histology Core in UCSD’s Moore’s Cancer Center and imaged with microscopy as previously reported^21^. H&E and PAS stained slide images were captured at 20X magnification for levels of peribronchial inflammation and PAS stained slides, respectively.

### ELISA

Samples stored at −20C were analyzed using IL-5 and IL-13 ELISA kits (R&D Systems, Minneapolis, MN) per the company’s instructions. Plates were read using a microplate reader model 680 (Bio-Rad Laboratories, Hercules, CA). ELISA data was analyzed using Excel and Graphpad Prism (San Diego, CA).

### ILC purification and RNA Sequencing

WT and *Rbm3*^*−/−*^ ILCs or WT ILCs based on ST2 and CD127 expression were sorted with the BD FACSAria II or the BD FACSAria Fusion at the UCSD Human Embryonic Stem Cell Core Facility. Lin^−^Thy1.2^+^ ILCs were sorted directly into TrizolLS. RNA-sequencing was performed at the La Jolla Institute. RNA-sequencing data of *Rbm3*^*−/−*^and WT ILCs was newly generated for this study and deposited in GEO database (GSE155330). RNA-sequencing of ILCs based ST2 and CD127 were previously reported^21^ and is deposited in GEO database (GSE136156).

Briefly, purified total RNA (≈5 ng) was amplified following the Smart-seq2 protocol^60,61^. mRNA was captured using poly-dT oligos and directly reverse-transcribed into full-length cDNA using the described template-switching oligo^60,61^. cDNA was amplified by PCR for 15 cycles and purified using AMPure XP magnetic bead (0.9:1 (vol:vol) ratio, Beckman Coulter). From this step, for each sample, 1 ng of cDNA was used to prepare a standard NextEra XT sequencing library (NextEra XT DNA library prep kit and index kits; Illumina). Barcoded Illumina sequencing libraries (NextEra; Illumina) were generated utilizing an automated platform (Biomek FXP, Beckman Coulter). Both whole-transcriptome amplification and sequencing library preparations were performed in a 96-well format to reduce assay-to-assay variability. Quality control steps were included to determine total RNA quality and quantity, the optimal number of PCR preamplification cycles, and fragment library size. The reference genome was mm10 (mouse genome). None of the samples failed quality controls. All of the samples were pooled at equimolar concentration, loaded, and sequenced on the Illumina Sequencing platform, HiSeq2500 (Illumina). Libraries were sequenced to obtain more than 10 million 50-bp single-end reads (HiSeq Rapid Run Cluster and SBS Kit V2; Illumina) mapping uniquely to mRNA reference.

### qPCR

RNA was reverse transcribed to cDNA using a Transcriptor First Strand cDNA Synthesis Kit (Roche) according to the manufacturer’s instructions. RT-PCR was performed using SYBR Green I Master (Roche) and RT-PCR–specific primers. The primer sequences (5′–3′) were as follows: *mIL-5* forward AAGAGAAGTGTGGCGAGGAGA; *mIL-5* reverse CACCAAGGAACTCTTGCAGGTAA; mIL-13 forward GAGCAACATCACACAAGACCAGA; mIL-13 reverse GCCAGGTCCACACTCCATA; *Cyslt1r* forward AACGAACTATCCACCTTCACC; *Cyslt1r* reverse AGCCTTCTCCTAAAGTTTCCAC; *L32* forward GAAACTGGCGGAAACCCA; and *L32* reverse GGATCTGGCCCTTGAACCTT. qPCR was completed using the Rbm3 transcript variant 2 (NM_001166409). Transcripts were measured relative to L32 using Roche LightCycler 480 (Roche Diagnostics)^58^. qPCR performed on CD127^+/-^ ST2^+/*−*^ Lin^−^Thy1.2^+^ lymphocytes utilized the following transcripts: *Rbm3* (Mm00812518_m1), *Tslpr* (Mm00497362_m1), *Cystlr1* (Mm02620326_s1), *Il13* (Mm00434204_m1), *Il17a* (Mm00439618_m1), *Cd127* (Mm01309416_m1), and *Thy1.2* (Mm00493681_m1). Transcripts were measured relative to *Hprt* using QuantStudio3 (Thermo Fisher Scientific).

### Flow Cytometry

For surface stains, 1 × 10^6^ lung and BAL cells were stained. For intracellular stains, 2 × 10^6^ lung cells were stained. Fc receptors were first blocked for 5 min using CD16/CD32 (Biolegend, San Diego, CA). Eosinophils were identified as CD11c^−^Siglec-F^+^ and neutrophils were identified as SiglecF^−^GR-1^+^; they were stained using CD45.2 (PerCP), Siglec-F (PE), GR-1(APC), and CD11c (FITC). ILCs were identified as Lineage^−^Thy1.2^+^ lymphocytes or Lineage^−^T1ST2^+^ lymphocytes and were stained using CD45.2 (PerCP), Thy1.2 (APC), T1ST2 (PE), and a lineage cocktail. The lineage cocktail (FITC) consisted of the BioLegend Lineage cocktail (consists of CD3e, Ly-6G/Ly-6C, CD11b, CD45R/B220, and TER-119), CD11c, NK1.1, CD5, FcεR1, TCRαβ, and TCRɣδ. The ILC subsets were stained with ST2 (APC) and CD127 (PE-Cy7). For nuclear intracellular staining, cells were permeabilized using the FoxP3 kit (ThermoFisher, Waltham, MA) after surface staining. Cells were stained with Ki-67 (PE or APC), RBM3, GATA3 (PE), and ID2 (PE).

For cytokine intracellular staining of lung cells in the 10-day challenge model, cells were cultured overnight with Golgi Plug (Fisher Scientific, Hampton, NH) at 500,000 cells per well. After surface staining for ILCs, cells were fixed and permeabilized using the BD fixation/permeabilization kit (BD Biosciences, La Jolla, CA) and stained for IL-5 (PE) or IL-13 (PE). For cytokine intracellular staining following the 7-day IL-33 and *Alternaria* challenge model, lung cells were cultured for 3 h with cell stimulation cocktail (ThermoFisher, Waltham, MA) at 1 × 10^6^ cells per well. After surface staining for ILCs, cells were fixed and permeabilized using the BD kit and stained for IL-5 (PE), IL-13 (PE), or IL17A (eFlour506). Lung cells stained for Bcl-2 expression were surfaced stained for ILCs, fixed and permeabilized with the BD kit, and stained with anti-Bcl-2 (PE-Cy7).

For human PBMC staining, ILC2s were sorted as Lineage^−^CRTH2^+^ lymphocytes. The lineage cocktail (FITC) consisted of antibodies for CD3, CD14, CD16, CD19, CD20, CD56, TCRγδ, CD4, CD11b, CD235a, and FcεRI. The polyclonal RBM3 antibody used in this study was raised in rabbits to the 14 c-terminal amino acids of RBM3, and affinity purified to the immunizing peptide. As described in prior work^62,63^, the affinity-purified anti-RBM3 antibody recognizes an ~17 kDa band corresponding to RBM3 on Western blots and selectively labels RBM3 in situ under a variety of fixation conditions. Flow Cytometry was performed using the BD Accuri for Fig. 1 and Supplemenatary Fig. 3b, otherwise, Acea Novocyte was used. Data was analyzed using FlowJo software (Tree Star, Ashland, OR). All antibodies were from Biolegend, ThermoFisher, or BD Biosciences.

### Immunofluorescence

For mouse airways, immunofluorescence for RBM3 was performed on naïve and *Alternaria* challenged airways as previously reported^64,65^. Briefly, lung samples were de-paraffinized by sequential placement in xylene and ethanol. Staining for RBM3 was performed with rabbit polyclonal antibody (PeproTech) at 1:1000 concentration. Tyramide Signal Amplification Kit #41 (Invitrogen) was used for fluorescent signal amplification with subsequent DAPI staining (Vector Laboratories). Lung airways were visualized with a DM2500 microscope (Leica Microsystems). Cell nuclei were stained with DAPI. Images were taken from at least 5 airways of at least 3 mice per group.

### ILC and PBMC cultures

WT and *Rbm3*^*−/−*^ Lin^−^Thy1.2^+^ ILCs were sorted using the BD FACSAria Fusion and BD FACSAria II sorters from UCSD’s Human Embryonic Stem Cell Core Facility. Collected ILCs were rested in 10 ng/mL IL-2 and IL-7 (R&D Systems, Minneapolis, MN) in T cell media (RPMI + 10% FBS, 1% glutamine, 0.1% BME, 1% pen/strep) for 48 h. ILCs were cultured in a 96-well plate at 40,000 cells per well. Pre-stimulation media was collected and stored in −80C for ELISA. ILCs were stimulated for 24 h with 15 ng/mL or 30 ng/mL IL-33 (R&D Systems, Minneapolis, MN) in T cell media. Post-stimulation supernatant was collected and stored at −80 C for ELISA analysis.

Human PBMCs were isolated from commercially purchased leukopacks (Allcells, Alameda, CA, USA). Human peripheral blood ILC2s were sorted as CD45^+^lin^−^CRTH2^+^ lymphocytes and cultured and treated with TSLP and IL-33 before being fixed with 4% PFA and processed for immunocytochemistry using a RBM3 antibody (1:2000) and a Cy3 secondary. Cells were also stained for DAPI. Images were taken at 20X. Immuno-positive elements were captured and analyzed by thresholding the intensity histogram in the Cy3 channel at 100. Data for objects of 50–175 pixels were included.

### Statistical analysis

Statistical analysis was performed with GraphPad Prism software (GraphPad Software, La Jolla, CA). P-values were obtained using the Mann-Whitney test, unpaired t-test, or one-way ANOVA and a P value of less than 0.05 was considered statistically significant such that *p < 0.05, **p < 0.01, ***p < 0.001.

### Reporting summary

Further information on research design is available in the Nature Research Reporting Summary linked to this article.

## Supplementary information

Supplementary Information

Description of Additional Supplementary Files

Supplementary Data

Reporting Summary

## Source data

Source Data

## Data Availability

Reagents for flow cytometry, qPCR, ELISA, allergen, and cytokines are detailed in the Supplementary Data file. RNA-sequencing data of *Rbm3*^*−/−*^ and WT ILCs was newly generated and deposited in GEO database (GSE155330). RNA-sequencing of ILCs based ST2 and CD127 was previously reported^21^ and is deposited in GEO database (GSE136156). There are no restrictions with obtaining unique biological materials which are available from the corresponding author upon reasonable request. Source data are provided with this paper.

## References

[1] Moro, K. et al. Innate production of TH2 cytokines by adipose tissue-associated c-Kit+Sca-1+ lymphoid cells. *Nature* **463**, 540–544 (2010). 10.1038/nature08636 20023630

[2] Kim, H. Y. et al. Interleukin-17-producing innate lymphoid cells and the NLRP3 inflammasome facilitate obesity-associated airway hyperreactivity. *Nat. Med.* **20**, 54–61 (2014). 10.1038/nm.3423 PMC3912313 24336249

[3] Chang, Y. J. et al. Innate lymphoid cells mediate influenza-induced airway hyper-reactivity independently of adaptive immunity. *Nat. Immunol.* **12**, 631–638 (2011). 10.1038/ni.2045 PMC3417123 21623379

[4] Hams, E. et al. IL-25 and type 2 innate lymphoid cells induce pulmonary fibrosis. *Proc. Natl Acad. Sci. USA* **111**, 367–372 (2014). 10.1073/pnas.1315854111 PMC3890791 24344271

[5] Artis, D. & Spits, H. The biology of innate lymphoid cells. *Nature* **517**, 293–301 (2015). 10.1038/nature14189 25592534

[6] Hurrell, B. P., Shafiei Jahani, P. & Akbari, O. Social Networking of group two innate lymphoid cells in allergy and asthma. *Front Immunol.* **9**, 2694 (2018). 10.3389/fimmu.2018.02694 PMC6256740 30524437

[7] Lambrecht, B. N., Hammad, H. & Fahy, J. V. The cytokines of asthma. *Immunity* **50**, 975–991 (2019). 10.1016/j.immuni.2019.03.018 30995510

[8] Gour, N. & Wills-Karp, M. IL-4 and IL-13 signaling in allergic airway disease. *Cytokine* **75**, 68–78 (2015). 10.1016/j.cyto.2015.05.014 PMC4532591 26070934

[9] Huang, Y. et al. IL-25-responsive, lineage-negative KLRG1(hi) cells are multipotential ‘inflammatory’ type 2 innate lymphoid cells. *Nat. Immunol.* **16**, 161–169 (2015). 10.1038/ni.3078 PMC4297567 25531830

[10] Cai, T. et al. IL-17-producing ST2(+) group 2 innate lymphoid cells play a pathogenic role in lung inflammation. *J. Allergy Clin. Immunol.* **143**, 229–244 e229 (2019). 10.1016/j.jaci.2018.03.007 PMC6170730 29625134

[11] Moro, K. et al. Interferon and IL-27 antagonize the function of group 2 innate lymphoid cells and type 2 innate immune responses. *Nat. Immunol.* **17**, 76–86 (2016). 10.1038/ni.3309 26595888

[12] Duerr, C. U. et al. Type I interferon restricts type 2 immunopathology through the regulation of group 2 innate lymphoid cells. *Nat. Immunol.* **17**, 65–75 (2016). 10.1038/ni.3308 PMC9135352 26595887

[13] Maric, J. et al. Prostaglandin E2 suppresses human group 2 innate lymphoid cell function. *J. Allergy Clin. Immunol.* **141**, 1761–1773 e1766 (2018). 10.1016/j.jaci.2017.09.050 PMC5929462 29217133

[14] Zhou, W. et al. Prostaglandin I2 signaling and inhibition of Group 2 innate lymphoid cell responses. *Am. J. Respir. Crit. Care Med.* **193**, 31–42 (2016). 10.1164/rccm.201410-1793OC PMC4731613 26378386

[15] Knolle, M. D. et al. MicroRNA-155 protects Group 2 innate lymphoid cells from apoptosis to promote Type-2 immunity. *Front. Immunol.* **9**, 2232 (2018). 10.3389/fimmu.2018.02232 PMC6189280 30356668

[16] Singh, P. B. et al. MicroRNA regulation of type 2 innate lymphoid cell homeostasis and function in allergic inflammation. *J. Exp. Med.* **214**, 3627–3643 (2017). 10.1084/jem.20170545 PMC5716040 29122948

[17] Pilotte, J., Dupont-Versteegden, E. E. & Vanderklish, P. W. Widespread regulation of miRNA biogenesis at the Dicer step by the cold-inducible RNA-binding protein, RBM3. *PLoS One* **6**, e28446 (2011). 10.1371/journal.pone.0028446 PMC3228759 22145045

[18] Stellato, C. et al. Coordinate regulation of GATA-3 and Th2 cytokine gene expression by the RNA-binding protein HuR. *J. Immunol.* **187**, 441–449 (2011). 10.4049/jimmunol.1001881 PMC5801757 21613615

[19] Gruber, A. R., Fallmann, J., Kratochvill, F., Kovarik, P. & Hofacker, I. L. AREsite: a database for the comprehensive investigation of AU-rich elements. *Nucleic Acids Res.* **39**, D66–D69 (2011). 10.1093/nar/gkq990 PMC3013810 21071424

[20] Hikichi, Y., Motomura, Y., Takeuchi, O. & Moro, K. Posttranscriptional regulation of ILC2 homeostatic function via tristetraprolin. *J. Exp. Med.* **218**, 1–12 (2021). 10.1084/jem.20210181 PMC8558840 34709349

[21] Cavagnero, K. J. et al. Unconventional ST2- and CD127-negative lung ILC2 populations are induced by the fungal allergen Alternaria alternata. *J. Allergy Clin. Immunol.* **144**, 1432–1435 e1439 (2019). 10.1016/j.jaci.2019.07.018 PMC6938690 31369800

[22] Sureban, S. M. et al. Translation regulatory factor RBM3 is a proto-oncogene that prevents mitotic catastrophe. *Oncogene* **27**, 4544–4556 (2008). 10.1038/onc.2008.97 PMC2677646 18427544

[23] Wong, J. J. et al. RBM3 regulates temperature sensitive miR-142-5p and miR-143 (thermomiRs), which target immune genes and control fever. *Nucleic Acids Res.* **44**, 2888–2897 (2016). 10.1093/nar/gkw041 PMC4824108 26825461

[24] Taylor, G. A. et al. A pathogenetic role for TNFα in the syndrome of cachexia, arthritis, and autoimmunity resulting from Tristetraprolin (TTP) Deficiency. *Immunity* **4**, 445–454 (1996). 10.1016/s1074-7613(00)80411-2 8630730

[25] Casolaro, V. et al. Posttranscriptional regulation of IL-13 in T cells: role of the RNA-binding protein HuR. *J. Allergy Clin. Immunol.* **121**, 853–859 e854 (2008). 10.1016/j.jaci.2007.12.1166 PMC2666917 18279945

[26] Doherty, T. A. et al. STAT6 regulates natural helper cell proliferation during lung inflammation initiated by Alternaria. *Am. J. Physiol. Lung Cell Mol. Physiol.* **303**, L577–L588 (2012). 10.1152/ajplung.00174.2012 PMC3469584 22865552

[27] Bartemes, K. R. et al. IL-33-responsive lineage- CD25+ CD44(hi) lymphoid cells mediate innate type 2 immunity and allergic inflammation in the lungs. *J. Immunol.* **188**, 1503–1513 (2012). 10.4049/jimmunol.1102832 PMC3262877 22198948

[28] Barlow, J. L. et al. IL-33 is more potent than IL-25 in provoking IL-13-producing nuocytes (type 2 innate lymphoid cells) and airway contraction. *J. Allergy Clin. Immunol.* **132**, 933–941 (2013). 10.1016/j.jaci.2013.05.012 23810766

[29] Seehus, C. R. et al. The development of innate lymphoid cells requires TOX-dependent generation of a common innate lymphoid cell progenitor. *Nat. Immunol.* **16**, 599–608 (2015). 10.1038/ni.3168 PMC4439271 25915732

[30] Zook, E. C. et al. The ETS1 transcription factor is required for the development and cytokine-induced expansion of ILC2. *J. Exp. Med* **213**, 687–696 (2016). 10.1084/jem.20150851 PMC4854726 27069114

[31] Halim, T. Y. F. et al. Retinoic-acid-receptor-related orphan nuclear receptor alpha is required for natural helper cell development and allergic inflammation. *Immunity* **37**, 463–474 (2012). 10.1016/j.immuni.2012.06.012 22981535

[32] Wong, S. H. et al. Transcription factor RORalpha is critical for nuocyte development. *Nat. Immunol.* **13**, 229–236 (2012). 10.1038/ni.2208 PMC3343633 22267218

[33] Mohapatra, A. et al. Group 2 innate lymphoid cells utilize the IRF4-IL-9 module to coordinate epithelial cell maintenance of lung homeostasis. *Mucosal. Immunol.* **9**, 275–286 (2016). 10.1038/mi.2015.59 PMC4698110 26129648

[34] He, M. X. & He, Y. W. A role for c-FLIP(L) in the regulation of apoptosis, autophagy, and necroptosis in T lymphocytes. *Cell Death Differ.* **20**, 188–197 (2013). 10.1038/cdd.2012.148 PMC3554340 23175183

[35] Mandal, M. et al. The BCL2A1 gene as a pre-T cell receptor-induced regulator of thymocyte survival. *J. Exp. Med.* **201**, 603–614 (2005). 10.1084/jem.20041924 PMC2213063 15728238

[36] Ambrosini, G., Adida, C. & Altieri, D. C. A novel anti-apoptosis gene, survivin, expressed in cancer and lymphoma. *Nat. Med.* **3**, 917–921 (1997). 10.1038/nm0897-917 9256286

[37] Marusawa, H. et al. HBXIP functions as a cofactor of survivin in apoptosis suppression. *EMBO J.* **22**, 2729–2740 (2003). 10.1093/emboj/cdg263 PMC156760 12773388

[38] von Moltke, J. et al. Leukotrienes provide an NFAT-dependent signal that synergizes with IL-33 to activate ILC2s. *J. Exp. Med.* **214**, 27–37 (2017). 10.1084/jem.20161274 PMC5206504 28011865

[39] Lund, S. J. et al. Leukotriene C4 potentiates IL-33–induced Group 2 innate lymphoid cell activation and lung inflammation. *J. Immunol.* **199**, 1096 (2017). 10.4049/jimmunol.1601569 PMC5531601 28667163

[40] Vivier, E. et al. Innate lymphoid cells: 10 years on. *Cell* **174**, 1054–1066 (2018). 10.1016/j.cell.2018.07.017 30142344

[41] Tait Wojno, E. D. & Artis, D. Emerging concepts and future challenges in innate lymphoid cell biology. *J. Exp. Med* **213**, 2229–2248 (2016). 10.1084/jem.20160525 PMC5068238 27811053

[42] Doherty, T. A. & Broide, D. H. Lipid regulation of group 2 innate lymphoid cell function: Moving beyond epithelial cytokines. *J. Allergy Clin. Immunol.* **141**, 1587–1589 (2018). 10.1016/j.jaci.2018.02.034 PMC5967253 29522852

[43] Matsuda, A. et al. Generation of mice deficient in RNA-binding motif protein 3 (RBM3) and characterization of its role in innate immune responses and cell growth. *Biochem Biophys. Res Commun.* **411**, 7–13 (2011). 10.1016/j.bbrc.2011.06.038 21684257

[44] Zhu, X., Buhrer, C. & Wellmann, S. Cold-inducible proteins CIRP and RBM3, a unique couple with activities far beyond the cold. *Cell Mol. Life Sci.* **73**, 3839–3859 (2016). 10.1007/s00018-016-2253-7 PMC5021741 27147467

[45] Kim, J. et al. Intratumorally establishing Type 2 innate lymphoid cells blocks tumor growth. *J. Immunol.* **196**, 2410–2423 (2016). 10.4049/jimmunol.1501730 26829987

[46] Colgan, S. P., Furuta, G. T. & Taylor, C. T. Hypoxia and Innate Immunity: Keeping Up with the HIFsters. *Annu Rev. Immunol.* **38**, 341–363 (2020). 10.1146/annurev-immunol-100819-121537 PMC7924528 31961750

[47] Ushio, A. & Eto, K. RBM3 expression is upregulated by NF-kappaB p65 activity, protecting cells from apoptosis, during mild hypothermia. *J. Cell Biochem.* **119**, 5734–5749 (2018). 10.1002/jcb.26757 29388696

[48] Gautier, V. et al. Extracellular IL-33 cytokine, but not endogenous nuclear IL-33, regulates protein expression in endothelial cells. *Sci. Rep.* **6**, 34255 (2016). 10.1038/srep34255 PMC5046127 27694941

[49] Petrova, T., Pesic, J., Pardali, K., Gaestel, M. & Arthur, J. S. C. p38 MAPK signalling regulates cytokine production in IL-33 stimulated Type 2 Innate Lymphoid cells. *Sci. Rep.* **10**, 3479 (2020). 10.1038/s41598-020-60089-0 PMC7044202 32103032

[50] Furusawa, J. et al. Critical role of p38 and GATA3 in natural helper cell function. *J. Immunol.* **191**, 1818–1826 (2013). 10.4049/jimmunol.1300379 PMC3765427 23851685

[51] Yang, H. J. et al. RNA-binding protein RBM3 prevents NO-induced apoptosis in human neuroblastoma cells by modulating p38 signaling and miR-143. *Sci. Rep.* **7**, 41738 (2017). 10.1038/srep41738 PMC5278414 28134320

[52] Krabbendam, L., Bal, S. M., Spits, H. & Golebski, K. New insights into the function, development, and plasticity of type 2 innate lymphoid cells. *Immunol. Rev.* **286**, 74–85 (2018). 10.1111/imr.12708 30294969

[53] Zhang, K. et al. Cutting edge: notch signaling promotes the plasticity of Group-2 innate lymphoid cells. *J. Immunol.* **198**, 1798–1803 (2017). 10.4049/jimmunol.1601421 PMC5321819 28115527

[54] Halim, T. Y. F. et al. Group 2 innate lymphoid cells are critical for the initiation of adaptive T helper 2 cell-mediated allergic lung inflammation. *Immunity* **40**, 425–435 (2014). 10.1016/j.immuni.2014.01.011 PMC4210641 24613091

[55] Gold, M. J. et al. Group 2 innate lymphoid cells facilitate sensitization to local, but not systemic, TH2-inducing allergen exposures. *J. Allergy Clin. Immunol.* **133**, 1142–1148 (2014). 10.1016/j.jaci.2014.02.033 24679471

[56] Rajput, C. et al. RORalpha-dependent type 2 innate lymphoid cells are required and sufficient for mucous metaplasia in immature mice. *Am. J. Physiol. Lung Cell Mol. Physiol.* **312**, L983–L993 (2017). 10.1152/ajplung.00368.2016 PMC5495952 28360114

[57] Lund, S. J. et al. Leukotriene C4 potentiates IL-33-induced Group 2 innate lymphoid cell activation and lung inflammation. *J. Immunol.* **199**, 1096–1104 (2017). 10.4049/jimmunol.1601569 PMC5531601 28667163

[58] Doherty, T. A. et al. Lung type 2 innate lymphoid cells express cysteinyl leukotriene receptor 1, which regulates TH2 cytokine production. *J. Allergy Clin. Immunol.* **132**, 205–213 (2013). 10.1016/j.jaci.2013.03.048 PMC3704056 23688412

[59] Zhou, B. et al. Thymic stromal lymphopoietin as a key initiator of allergic airway inflammation in mice. *Nat. Immunol.* **6**, 1047–1053 (2005). 10.1038/ni1247 16142237

[60] Picelli, S. et al. Full-length RNA-seq from single cells using Smart-seq2. *Nat. Protoc.* **9**, 171–181 (2014). 10.1038/nprot.2014.006 24385147

[61] Rosales, S. L. et al. A sensitive and integrated approach to profile messenger RNA from samples with low cell numbers. *Methods Mol. Biol.* **1799**, 275–301 (2018). 10.1007/978-1-4939-7896-0_21 29956159

[62] Pilotte, J., Cunningham, B. A., Edelman, G. M. & Vanderklish, P. W. Developmentally regulated expression of the cold-inducible RNA-binding motif protein 3 in euthermic rat brain. *Brain Res.* **1258**, 12–24 (2009). 10.1016/j.brainres.2008.12.050 19150436

[63] Dresios, J. et al. Cold stress-induced protein Rbm3 binds 60S ribosomal subunits, alters microRNA levels, and enhances global protein synthesis. *Proc. Natl Acad. Sci. USA* **102**, 1865–1870 (2005). 10.1073/pnas.0409764102 PMC548588 15684048

[64] Doherty, T. A. et al. Alternaria induces STAT6-dependent acute airway Eosinophilia and Epithelial FIZZ1 expression that promotes airway fibrosis and epithelial thickness. *J. Immunol.* **188**, 2622–2629 (2012). 10.4049/jimmunol.1101632 PMC3294141 22327070

[65] Pilotte, J. et al. Morphoregulatory functions of the RNA-binding motif protein 3 in cell spreading, polarity and migration. *Sci. Rep.* **8**, 7367 (2018). 10.1038/s41598-018-25668-2 PMC5943363 29743635

