# Author Correction: RNA-binding protein RBM3 intrinsically suppresses lung innate lymphoid cell activation and inflammation partially through CysLT1R

**DOI:** 10.1038/s41467-026-78083-x

**Published:** 2026-09-25

**Authors:** Jana H. Badrani, Allyssa N. Strohm, Lee Lacasa, Blake Civello, Kellen Cavagnero, Yung-An Huang, Michael Amadeo, Luay H. Naji, Sean J. Lund, Anthea Leng, Hyojoung Kim, Rachel E. Baum, Naseem Khorram, Monalisa Mondal, Grégory Seumois, Julie Pilotte, Peter W. Vanderklish, Heather M. McGee, Taylor A. Doherty

**Affiliations:** 1 Divison of Rheumatology, Allergy and Immunology, Department of Medicine, University of California San Diego, La Jolla, CA USA; 2 Veterans Affairs San Diego Health Care System, La Jolla, CA USA; 3 Department of Microbiology and Immunology, College of Medicine, Chang Gung University, Taoyuan, Taiwan; 4 La Jolla Institute, La Jolla, CA USA; 5 The Scripps Research Institute, La Jolla, CA USA; 6 NOMIS Center for Immunobiology and Microbial Pathogenesis, Salk Institute, La Jolla, CA USA; 7 Departments of Radiation Oncology and Immuno-Oncology, City of Hope, Duarte, CA USA; 8 Department of Molecular Medicine, La Jolla, CA USA

**Keywords:** Asthma, Innate lymphoid cells, Mucosal immunology

Correction to: *Nature Communications* https://doi.org/10.1038/s41467-022-32176-5, published online 30 July 2022

In the version of this article initially published, the last name of Yung-An Huang was spelled incorrectly (Haung) and is now amended in the HTML and PDF versions of the article.

