# Supplementary material for: RNA-binding protein RBM3 intrinsically suppresses lung innate lymphoid cell activation and inflammation partially through CysLT1R
Source: Nat Commun. 2022 Jul 30;13:4435. doi: 10.1038/s41467-022-32176-5 (PMC9338970; doi:10.1038/s41467-022-32176-5)
Supplement: Supplementary file 1 — Supplementary Information [file 41467_2022_32176_MOESM1_ESM.pdf]

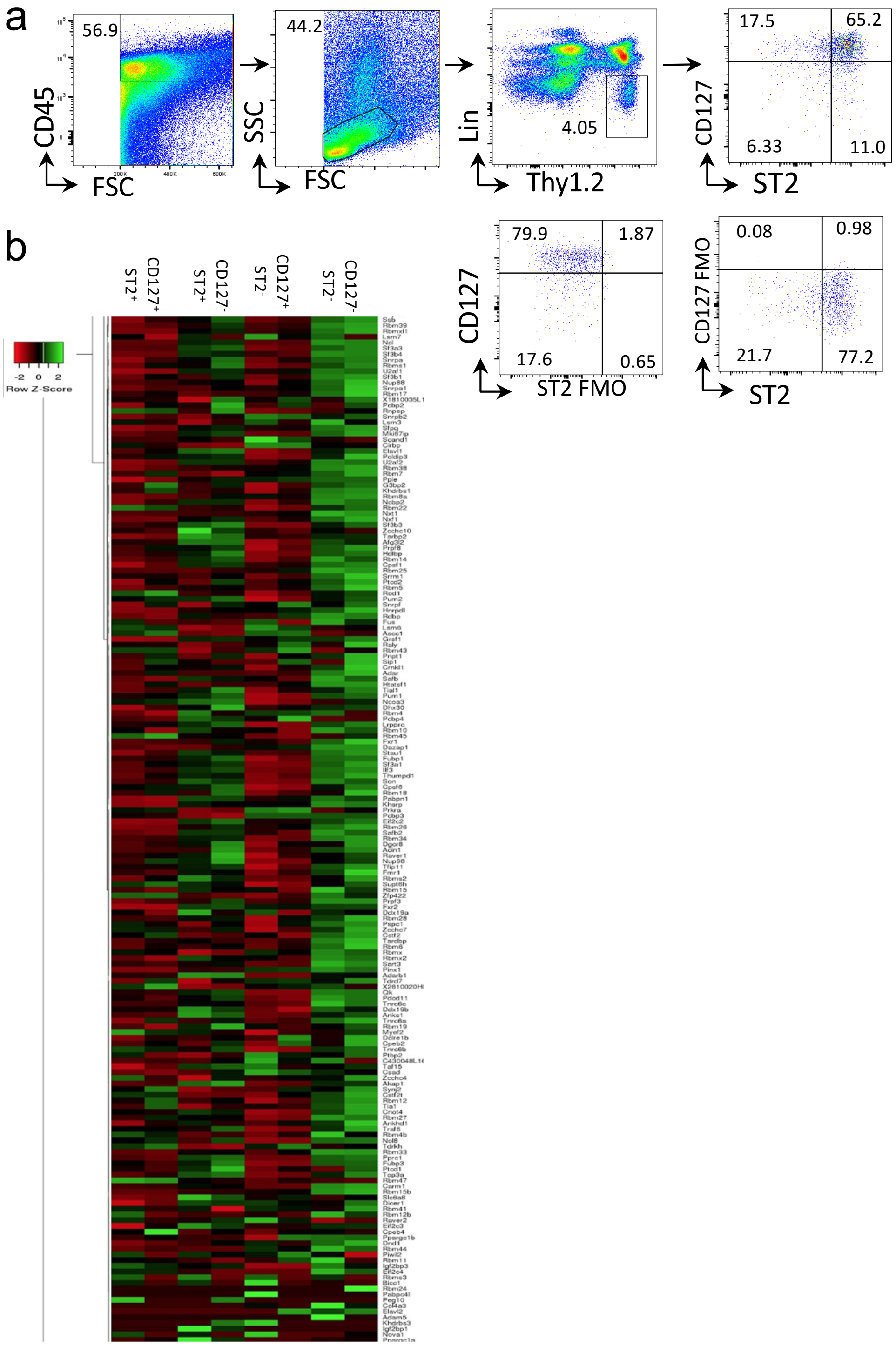

Supplementary Figure 1. Representative gating strategy for ILC subsets collected for bulk RNA-seq analysis (a) ILCs were gated as Lineage$^-$, Thy1.2$^+$ lymphocytes. Subsets were identified by their expression of ST2 and CD127. Gating was based on FMOs. (b) Wild-type mice were challenged 4 times with 50µg *Alternaria* over 10 days and four ILC subsets (CD127$^+$ST2$^+$, CD127$^-$ST2$^+$, CD127$^+$ST2$^-$, CD127$^-$ST2$^-$) were sorted and collected for RNA-seq. Heatmap showing relative expression of 207 RBPs in the four sorted ILC subtypes using Centroid linkage and Manhattan clustering analysis.

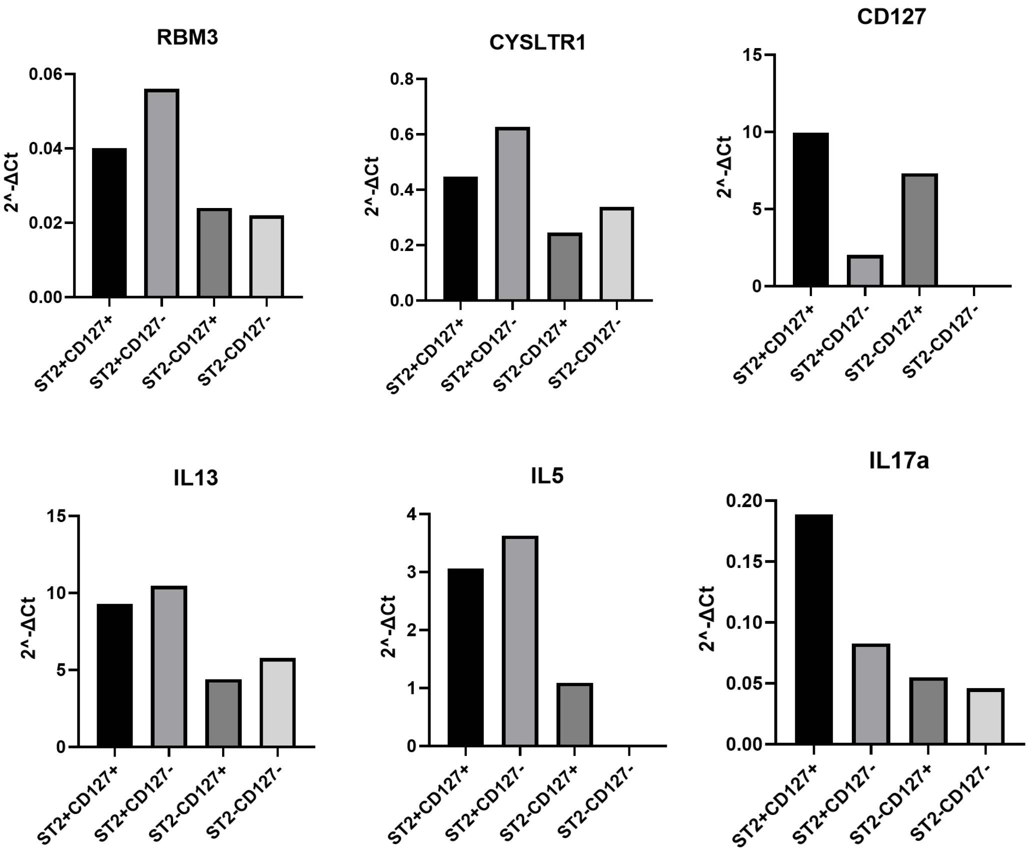

Supplementary Figure 2. Wild-type mice were challenged with *Alternaria* four times over 10 days. $Lin^{-}Thy1.2^{+}$ ILCs were FACS-sorted based on ST2 and CD127 expression. mRNA levels for RBM3 and select surface receptors and cytokines were compared across the four subsets.

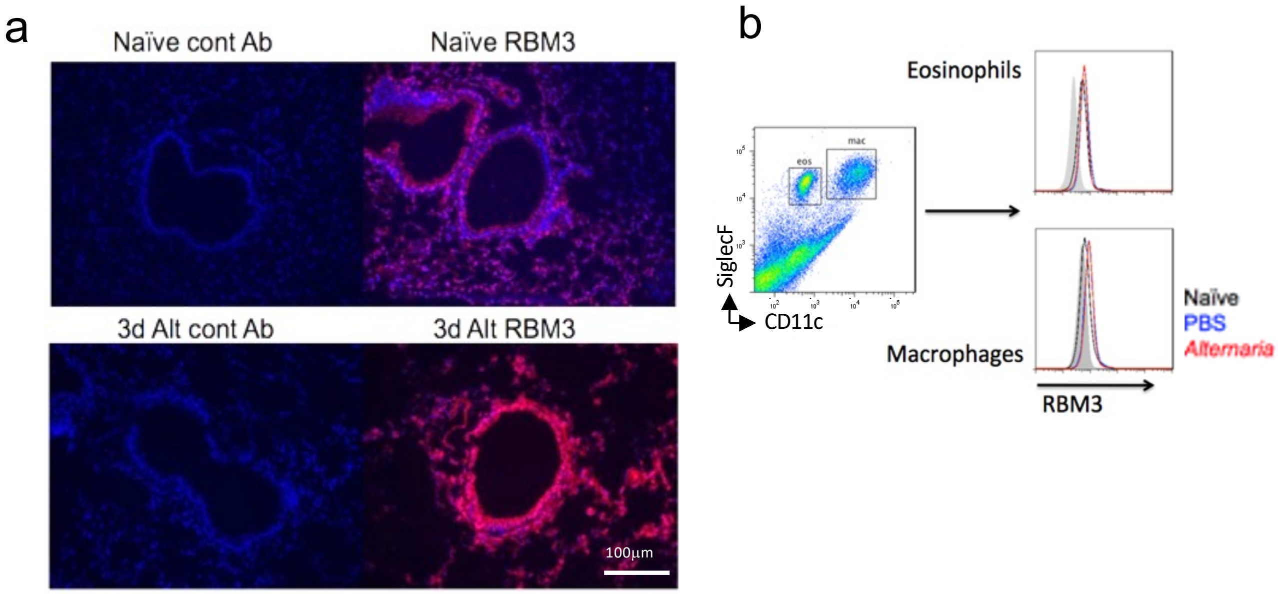

Supplementary Figure 3. RBM3 is expressed on multiple immune and non-immune cells within the airway. (a) RBM3 expression on macrophages and eosinophils from naive, PBS challenged, or *Alternaria* challenged lung. Black = Naive, Blue = PBS challenged, Red = *Alternaria* challenged (24 hour). (b) Immunofluorescence of RBM3 expression in airways of naïve and *Alternaria* challenged mice. Cell nuclei were stained with DAPI. Images were taken from at least 5 airways of at least 3 mice.

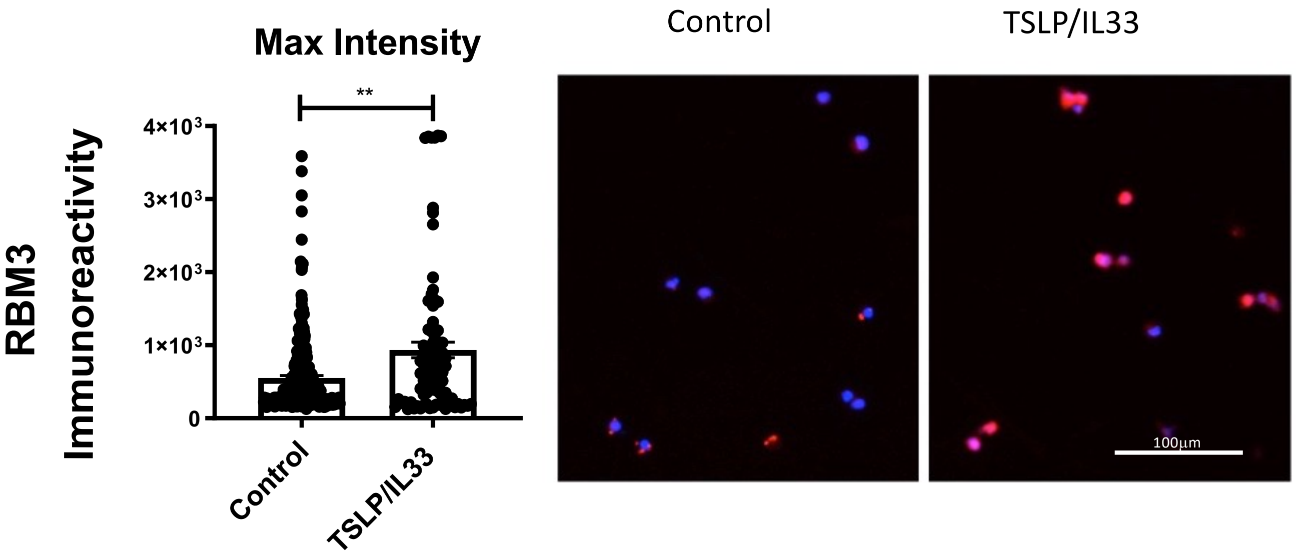

Supplementary Figure 4. RBM3 expression is upregulated in human blood ILC2s after stimulation with IL33 and TSLP. Human ILC2s were cultured and treated with TSLP and IL-33 before being fixed with 4% PFA and processed for immunocytochemistry using an RBM3 antibody (at 1:2000) and a Cy3 secondary antibody. Cells were also stained for DAPI. Images were taken at 20X. Immuno-positive elements were captured and analyzed by thresholding the intensity histogram in the Cy3 channel at 100. Maximum intensity was quantified after 24 hours of stimulation ($p = 0.0057$). Data for objects of 50-175 pixels were included ($n = 283$ for controls and $n = 87$ for TSLP/IL-33 group). Immunostaining shown after 72 hours of stimulation. Mann-Whitney Test, two-tailed. ** $p < 0.01$. Data are presented as mean values +/- SEM.

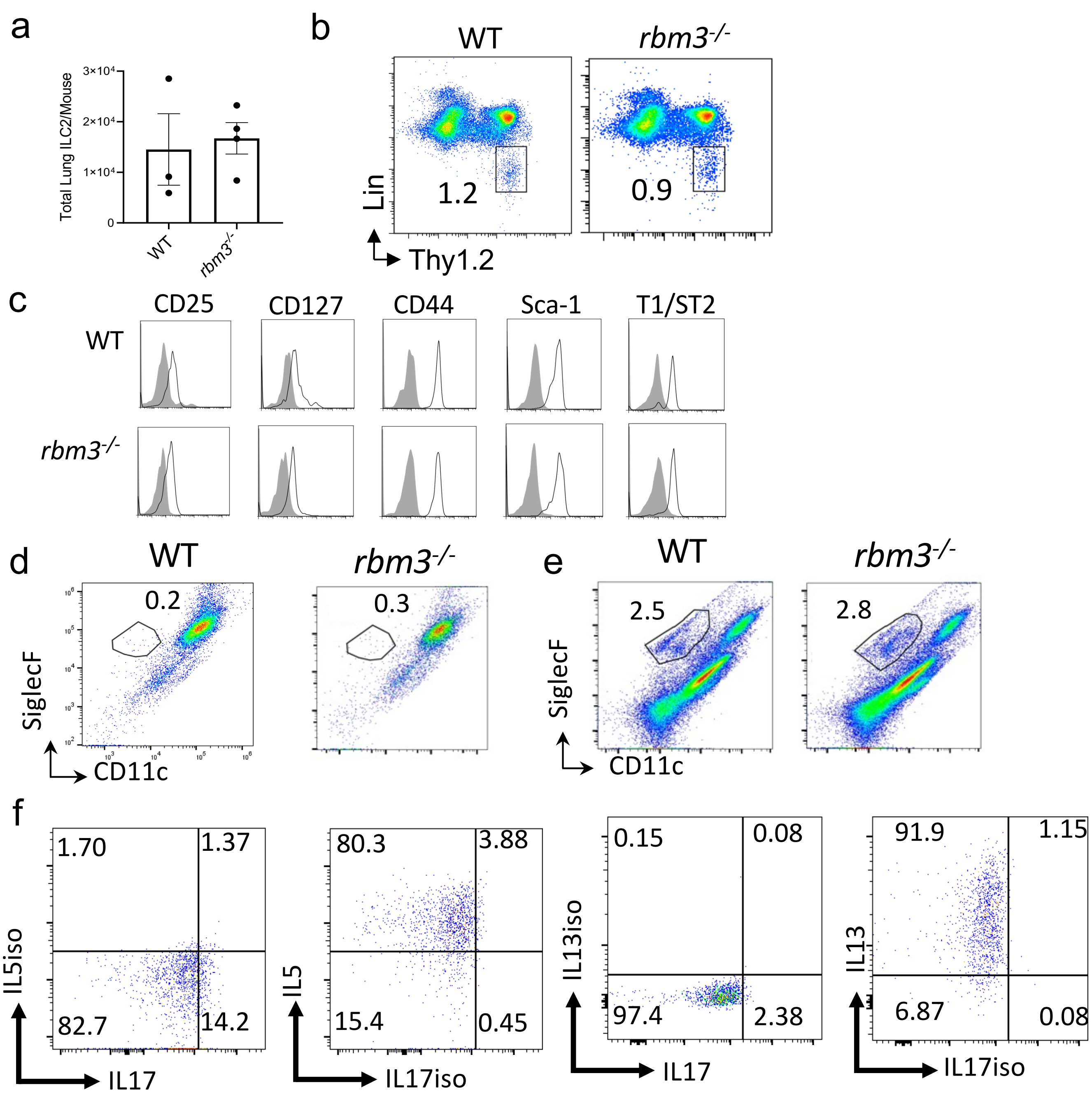

Supplementary Figure 5. Naive *rbm3*$^{-/-}$ mice do not have significant changes in ILC2 numbers, ILC2 surface marker expression, or eosinophilia. Naive wild-type and *rbm3*$^{-/-}$ lungs were stained for ILCs and eosinophils. (a) Total lung ILCs (p = 0.7638). Data representative of 3-4 mice. Unpaired t-test, two-tailed. (b) Representative FACS plots of Lin^{-}Thy1.2^{+} ILCs. (c) Histograms of surface marker expression on naive lung ILCs. Grey = Isotype control. Representative FACS plots of naïve (d) BAL eosinophils and (e) lung eosinophils are shown. (f) Isotype staining for IL-5 vs. IL-17 and IL-5 vs. IL-13 in Lin^{-}Thy1.2^{+} lymphocytes from *Alternaria*-challenged mice in Fig. 3E. Data are presented as mean values +/- SEM.

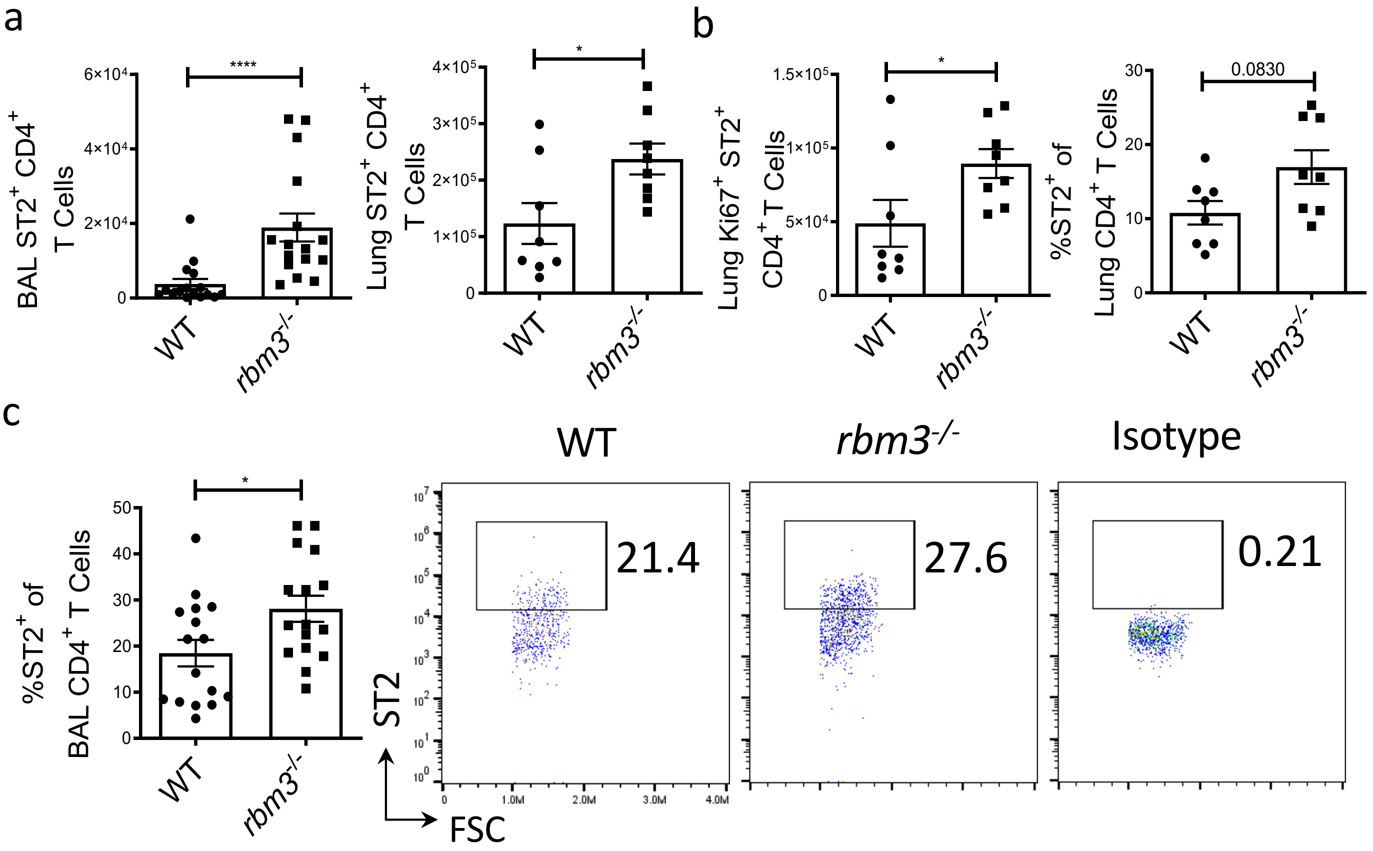

Supplementary Figure 6. *Alternaria*-challenged *rbm3$^{-/-}$* mice show an increased Th2 cell response. Mice were challenged with 20µg and 10µg *Alternaria* for 10 days. (a) Total BAL ($p < 0.0001$) and lung ($p = 0.0379$) Th2 cells. Th2 cells were identified as $CD4^{+}T1ST2^{+}$ lymphocytes. Data representative of 16 mice. (b) Total Ki-67 expressing lung Th2 cells ($p = 0.0499$) and percentage of T1ST2 expression ($p = 0.0830$) on lung $CD4^{+}$ T cells. Data representative of 16 paired lungs (generating 8 samples). (c) Percentage of T1ST2 expression on BAL $CD4^{+}$ T cells ($p = 0.0280$). Representative FACS plots of T1ST2 percentages and isotype control. Data representative of 16 mice. * $p < 0.05$, ** $p < 0.01$, *** $p < 0.001$, **** $p < 0.0001$, Mann Whitney test, two-tailed. Data are presented as mean values +/- SEM.

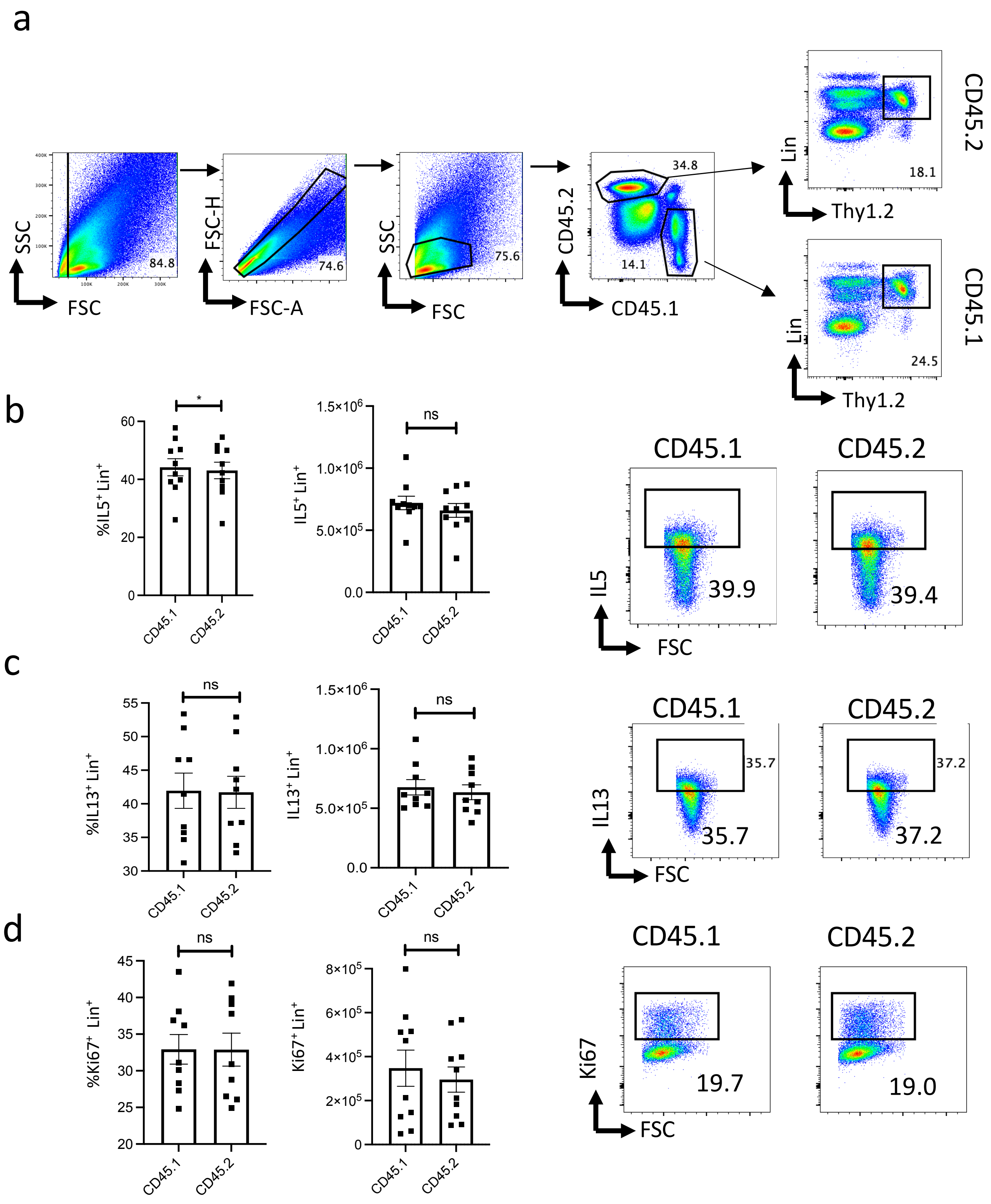

Supplementary Figure 7. Lineage^{+}Thy1.2^{+} cells show no differences in type 2 cytokine production or Ki67 expression in mixed bone marrow chimeras (a) Mixed bone marrow chimera mice were challenged 3 times over 7 days with 20µg *Alternaria*. FACS plots are representative of 2 independent experiments (5 mice each) with biological controls. Adaptive cells gated as lineage^{+}Thy1.2^{+}. (b) Percent (p=0.0151) and total IL5 (p=0.1896) expressing lineage^{+} cells from WT vs. *rbm3*$^{-/-}$ mice. FACS plots of IL5 percentages. (c) Percent (p=0.6466) and total IL13 (p=0.3425) expressing lineage^{+} cells from WT vs. *rbm3*$^{-/-}$ mice. Representative FACS plots of IL13 percentages. (d) Percent (p=0.9844) and total Ki67 (p=0.1374) expressing lineage^{+} cells from WT vs *rbm3*$^{-/-}$ mice. Representative FACS plots of Ki67 percentages. Paired t-Test, two-tailed. *p<0.05, **p<0.005, ***p<0.0005.

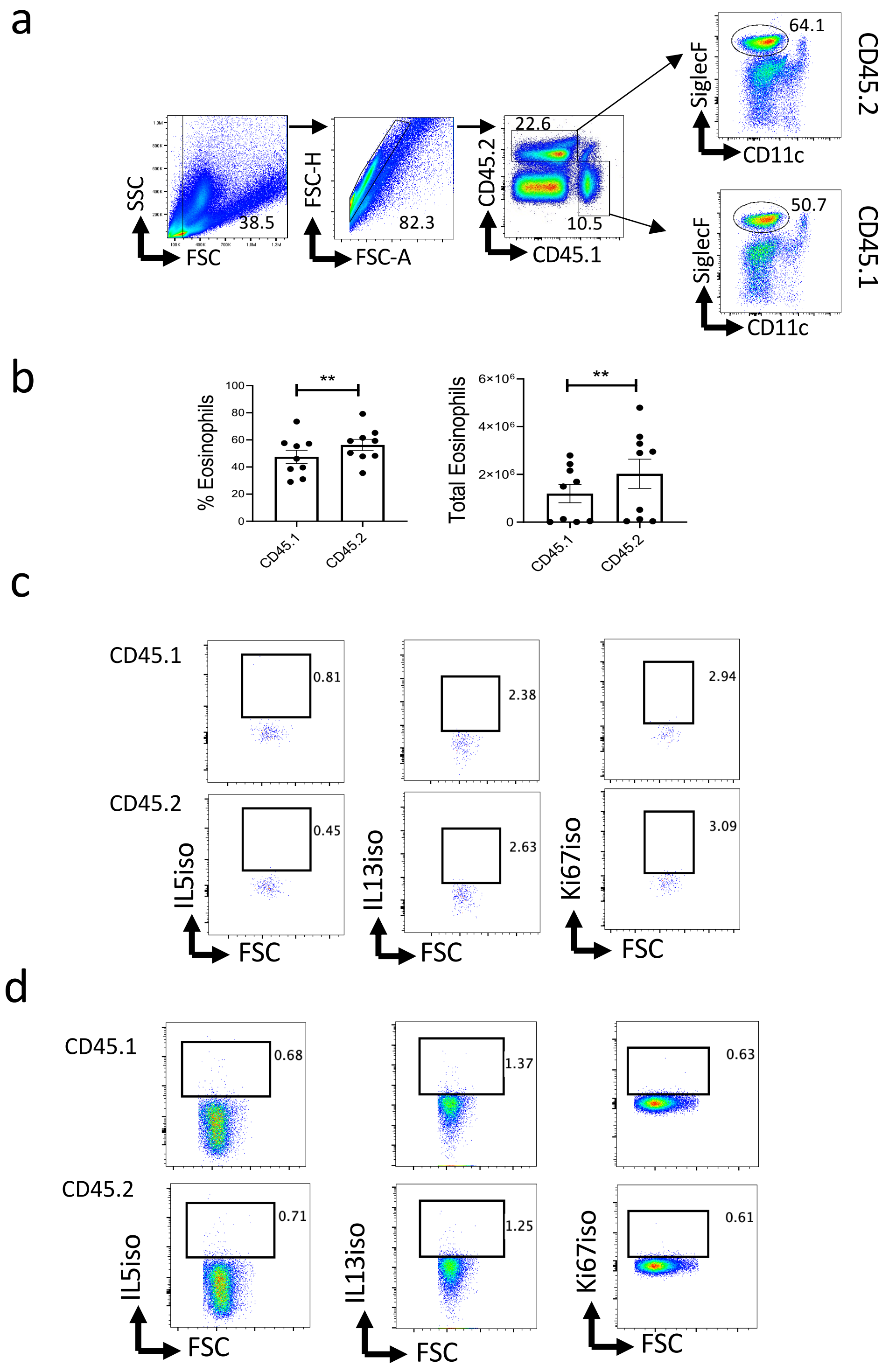

Supplementary Figure 8. Intrinsic differences in eosinophils from *rbm3*$^{-/-}$ mixed chimeric mice and lymphocyte isotypes. Airway eosinophils were gated (a) on CD45.1 or CD45.2 (*rbm3*$^{-/-}$) and assessed for eosinophils as Siglec-F^{+}CD11c^{-} (b), p=0.0023 and 0.0093, respectively. Paired t-Test. Representative of 2 independent experiments (5 mice each, n=9 as one mouse was omitted). **p<0.005. Isotype staining for IL-5, IL-13, Ki-67 from CD45.1 and CD45.2 ILCs (c) and Th2 cells (d). Data are presented as mean values +/- SEM.

a

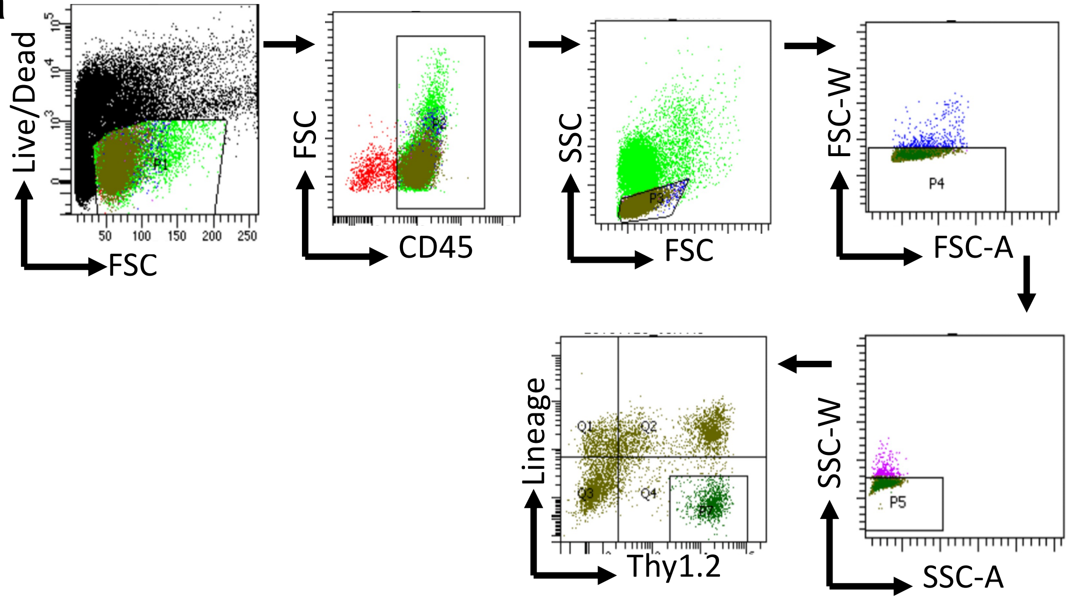

b

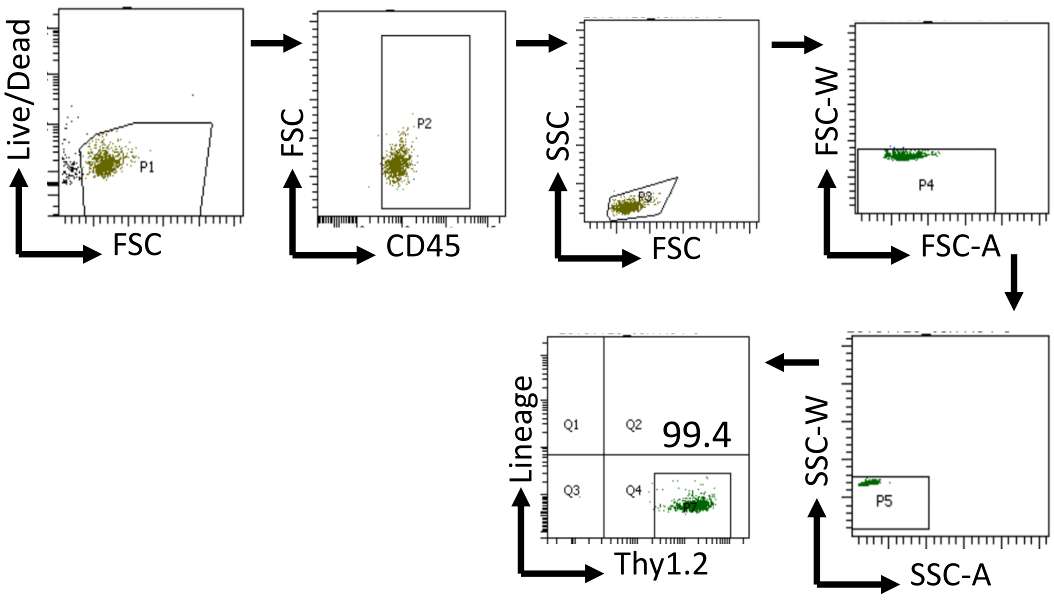

Supplementary Figure 9. Purity of ILCs from *Alternaria*-challenged WT and *rbm3*$^{-/-}$ mice FACS-sorted for RNA-seq analysis. WT and *rbm3*$^{-/-}$ mice were challenged with 25 µg *Alternaria* three times over 7 days. Lin^{-}Thy1.2^{+} ILCs were FACS-sorted and bulk RNA-sequenced. (a) Plots before sorting for Lin^{-}Thy1.2^{+} ILCs. (b) Post-sort plot demonstrating population purity.

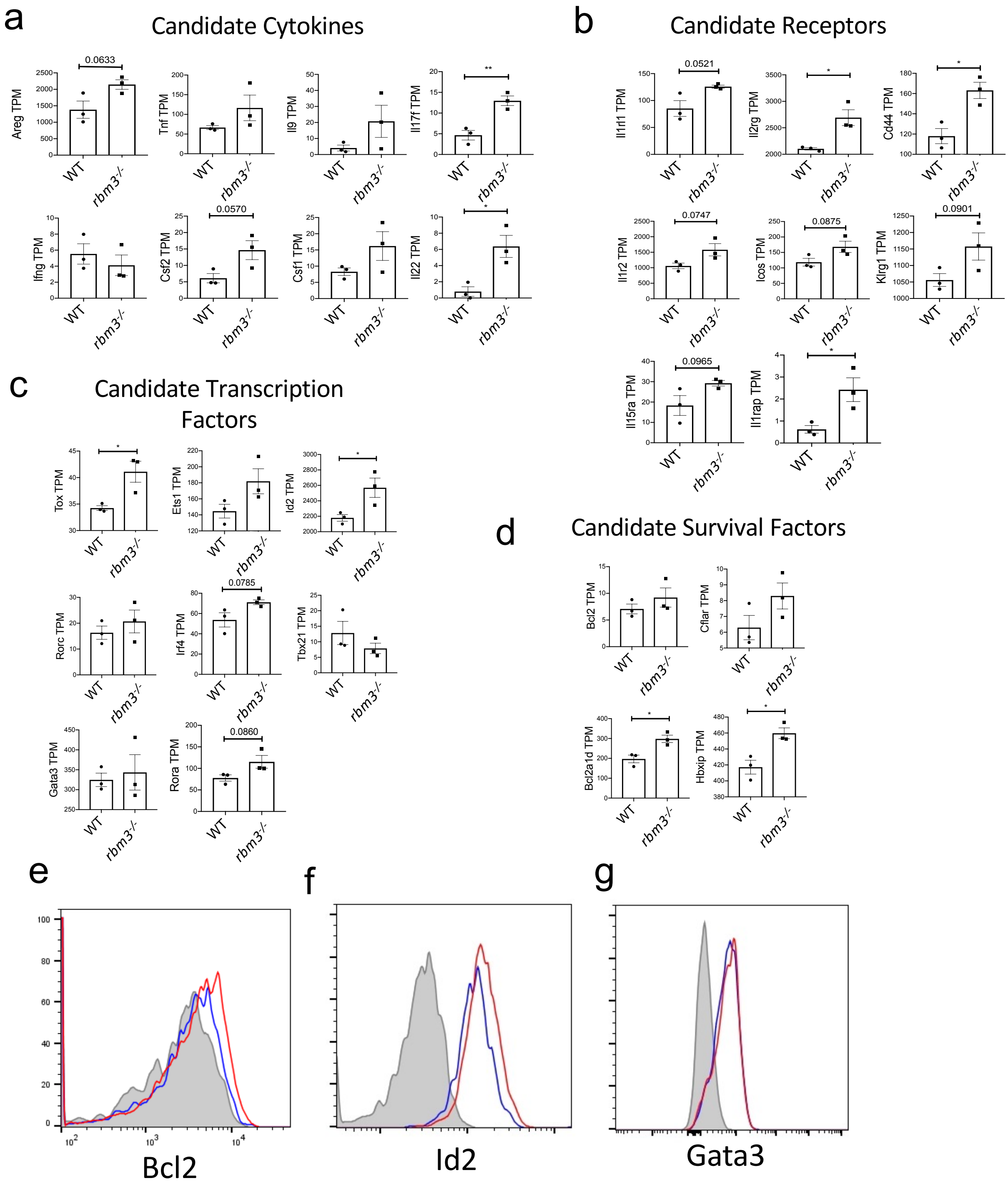

Supplementary Figure 10. FACS-sorted *Alternaria*-challenged WT and *Rbm3*$^{-/-}$ ILCs demonstrate differentially expressed transcriptomes. WT and *rbm3*$^{-/-}$ mice were challenged with 25 µg *Alternaria* three times over 7 days. Lin^{-}Thy1.2^{+} ILCs were FACS-sorted and bulk RNA-sequenced. Data representative of three mice per group. (a) Transcripts per million (TPMs) of select cytokine transcripts. Unpaired t-Test, two-tailed. (b) TPMs of select surface marker transcripts. Unpaired t-Test, two-tailed. (c) TPMs of select transcription factor transcripts. Unpaired t-Test, two-tailed. (d) TPMs of select transcripts involved in apoptosis. Unpaired t-test, two-tailed. WT and *rbm3*$^{-/-}$ mice were challenged with 25 µg *Alternaria* three times over 7 days. Data representative of 4 mice per group. (e) Histogram of Bcl2 expression. (f) Histogram of Id2 expression. (g) Histogram of GATA3 expression. Blue = WT. Red = *Rbm3*$^{-/-}$ Grey = isotype control. Unpaired t-Test, two-tailed. * $p < 0.05$, ** $p < 0.01$, *** $p < 0.001$. Data are presented as mean values +/- SEM.

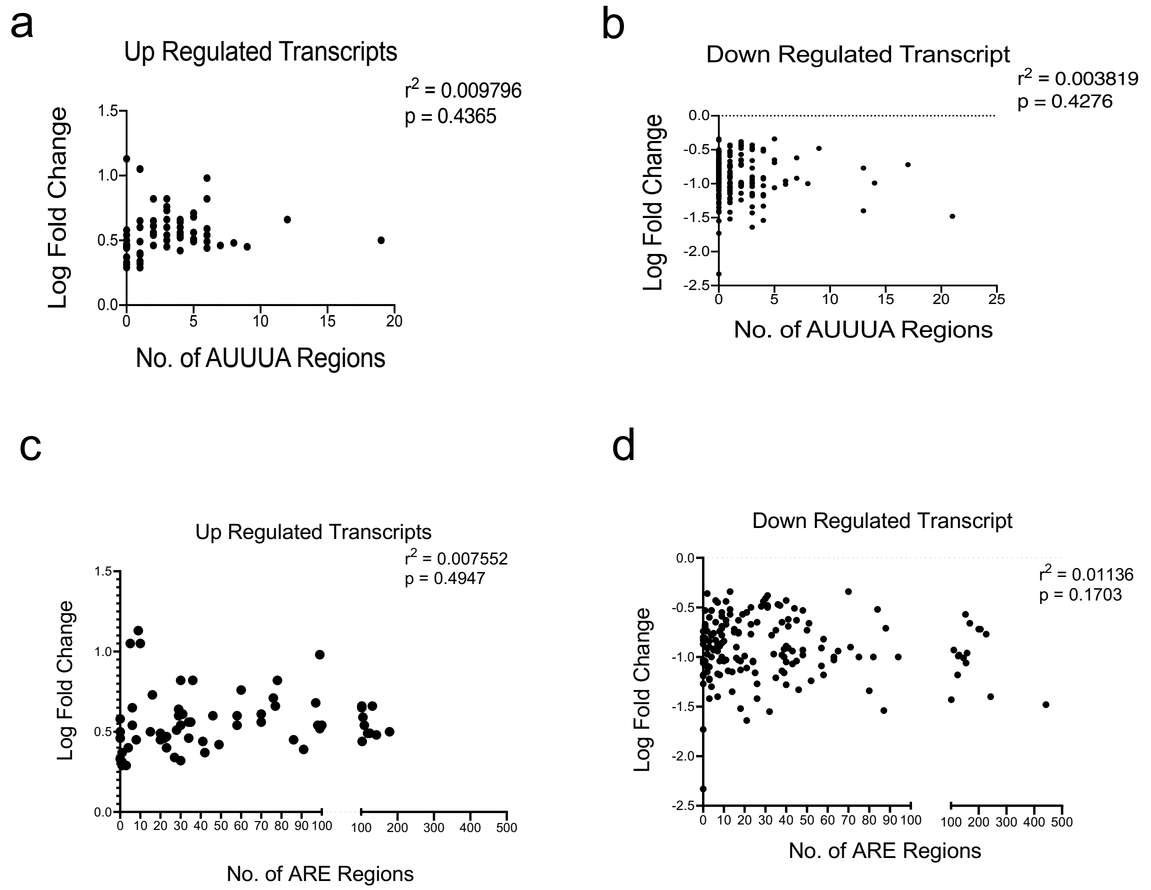

Supplementary Figure 11. Log fold change of differentially expressed transcripts of WT and *rbm3*$^{-/-}$ ILCs compared to the number of AUUUA or total number of ARE regions. WT and *rbm3*$^{-/-}$ mice were challenged with 25 µg *Alternaria* three times over 7 days. Lin$^-$Thy1.2$^+$ ILCs were FACS-sorted and bulk RNA-sequenced. RBPs were divided into two categories based on whether they were up-regulated or down-regulated. Utilizing an AUUUA and ARE database (http://nibiru.tbi.univie.ac.at/AREsite2/welcome), the total number of regions were determined and compared to the log fold change in transcriptional regulation. Comparison of (a) up-regulated and (b) down-regulated transcripts to their total number of AUUUA regions. Comparison of (c) up-regulated and (d) down-regulated transcripts to their total number of ARE regions. Pearson correlation, two-tailed.
