# Supplementary material for: RNA-binding protein RBM3 intrinsically suppresses lung innate lymphoid cell activation and inflammation partially through CysLT1R
Source: Nat Commun. 2022 Jul 30;13:4435. doi: 10.1038/s41467-022-32176-5 (PMC9338970; doi:10.1038/s41467-022-32176-5)
Supplement: Supplementary file 2 — Description of Additional Supplementary Files [file 41467_2022_32176_MOESM2_ESM.docx]

Description of additional supplementary files

**Supplementary Data.**  Reagent list that includes flow cytometry antibodies, ELISA kits, qPCR primers, allergen extract and cytokines. Dilutions, clones and catalog numbers listed where applicable.
