# Supplementary material for: RNA-binding protein RBM3 intrinsically suppresses lung innate lymphoid cell activation and inflammation partially through CysLT1R
Source: Nat Commun. 2022 Jul 30;13:4435. doi: 10.1038/s41467-022-32176-5 (PMC9338970; doi:10.1038/s41467-022-32176-5)
Supplement: Supplementary file 4 — Reporting Summary [file 41467_2022_32176_MOESM4_ESM.pdf]

nature research

Corresponding author(s): Taylor A. Doherty, M.D.

Last updated by author(s): May 17, 2022

Reporting Summary

Nature Research wishes to improve the reproducibility of the work that we publish. This form provides structure for consistency and transparency in reporting. For further information on Nature Research policies, see our Editorial Policies and the Editorial Policy Checklist.

Statistics

For all statistical analyses, confirm that the following items are present in the figure legend, table legend, main text, or Methods section.

n/a	Confirmed	
☐	☒	The exact sample size (*n*) for each experimental group/condition, given as a discrete number and unit of measurement
☐	☒	A statement on whether measurements were taken from distinct samples or whether the same sample was measured repeatedly
☐	☒	The statistical test(s) used AND whether they are one- or two-sided<br>*Only common tests should be described solely by name; describe more complex techniques in the Methods section.*
☒	☐	A description of all covariates tested
☒	☐	A description of any assumptions or corrections, such as tests of normality and adjustment for multiple comparisons
☒	☐	A full description of the statistical parameters including central tendency (e.g. means) or other basic estimates (e.g. regression coefficient) AND variation (e.g. standard deviation) or associated estimates of uncertainty (e.g. confidence intervals)
☐	☒	For null hypothesis testing, the test statistic (e.g. *F*, *t*, *r*) with confidence intervals, effect sizes, degrees of freedom and *P* value noted<br>*Give P values as exact values whenever suitable.*
☒	☐	For Bayesian analysis, information on the choice of priors and Markov chain Monte Carlo settings
☒	☐	For hierarchical and complex designs, identification of the appropriate level for tests and full reporting of outcomes
☐	☒	Estimates of effect sizes (e.g. Cohen's *d*, Pearson's *r*), indicating how they were calculated

Our web collection on statistics for biologists contains articles on many of the points above.

Software and code

Policy information about availability of computer code

Data collection	There was no custom computer code or algorithm used that was not reported to collect data.
Data analysis	GraphPad Prism was used for data analysis.

For manuscripts utilizing custom algorithms or software that are central to the research but not yet described in published literature, software must be made available to editors and reviewers. We strongly encourage code deposition in a community repository (e.g. GitHub). See the Nature Research guidelines for submitting code & software for further information.

Data

Policy information about availability of data

All manuscripts must include a data availability statement. This statement should provide the following information, where applicable:

- Accession codes, unique identifiers, or web links for publicly available datasets
- A list of figures that have associated raw data
- A description of any restrictions on data availability

Source data for all figures are provided with this paper as a supplement file. RNA-sequencing data of Rbm3-/- and WT ILCs is deposited in GEO database (GSE155330, https://www.ncbi.nlm.nih.gov/geo/query/acc.cgi?acc=GSE155330). ILC RNA-sequencing of ILCs based ST2 and CD127 are deposited in GEO database (GSE136156, https://www.ncbi.nlm.nih.gov/geo/query/acc.cgi?acc=GSE136156).

Field-specific reporting

Please select the one below that is the best fit for your research. If you are not sure, read the appropriate sections before making your selection.

- [x] Life sciences
- [ ] Behavioural & social sciences
- [ ] Ecological, evolutionary & environmental sciences

For a reference copy of the document with all sections, see nature.com/documents/nr-reporting-summary-flat.pdf

Life sciences study design

All studies must disclose on these points even when the disclosure is negative.

Sample size	Sample size was chosen based previous studies with mouse models of asthma showing statistical significance in asthma model and ILC endpoints. Individual data points shown.
Data exclusions	No data was excluded.
Replication	All mouse work was replicated and shown.
Randomization	N/A
Blinding	N/A

Reporting for specific materials, systems and methods

We require information from authors about some types of materials, experimental systems and methods used in many studies. Here, indicate whether each material, system or method listed is relevant to your study. If you are not sure if a list item applies to your research, read the appropriate section before selecting a response.

Materials & experimental systems

n/a	Involved in the study	
	x	Antibodies
x		Eukaryotic cell lines
x		Palaeontology and archaeology
	x	Animals and other organisms
x		Human research participants
x		Clinical data
x		Dual use research of concern

Methods

n/a	Involved in the study	
x		ChIP-seq
	x	Flow cytometry
x		MRI-based neuroimaging

Antibodies

Antibodies used

Mouse andtibodies
Vendor Antibody Clone Catalog Number Dilution
Biolegend Purified anti-mouse CD16/32 93 101302 1:50
Biolegend FITC Lineage anti-mouse 145-2C11 ; RB6-8C5 ; RA3-6B2 ; Ter-119 ; M1/70 133302 1:40
Biolegend FITC anti-mouse CD11c Antibody N418 117306
Biolegend FITC anti-mouse NK-1.1 Antibody PK136 108706
Biolegend FITC anti-mouse CD5 53-7.3 100606
Biolegend FITC anti-mouse FcεRIα Antibody MAR-1. 134306
Biolegend FITC anti-mouse TCR β chain Antibody H57-597 109206
Biolegend FITC anti-mouse TCR γ/δ GL3 118106
Thermo Fisher CD45.1 Monoclonal Antibody (A20), eFluor 450, eBioscience™ A20 48-0453-80 1:40
Thermo Fisher CD45.2 Monoclonal Antibody (104), APC-eFluor 780, eBioscience™ 104 47-0454-82 1:20
Biolegend PerCP/Cyanine5.5 anti-mouse CD45.2 104 109828 1:50
Thermo Fisher CD90.2 (Thy-1.2) Monoclonal Antibody (53-2.1), eFluor 450 53-2.1 48-0902-82 1:50
Biolegend PE/Cyanine7 anti-mouse CD127 (IL-7Rα) Antibody A7R34 135014 1:50
Biolegend APC anti-mouse IL-33Rα (IL1RL1, ST2) 100 µg DIH9 145306 1:50
BD Biosciences PE anti-mouse SiglecF E502440 552126 1:50
Biolegend APC anti-mouse Ly6G-Ly6C (GR1) RB6-8C5 108412 1:50
Thermo Fisher Ki-67 Monoclonal Antibody (SolA15), PE SolA15 12-5698-82 1:50
Thermo Fisher Anti-Hu/Mo Gata3 PE TWAJ 12-9966-42 1:20
Thermo Fisher ROR gamma (t) Monoclonal Antibody (B2D), PE, eBioscience™ B2D 12-6981-82 1:20
Biolegend PE anti-T-bet Antibody 4B10 644810 1:20

Biolegend PE anti-mouse/human IL-5 TRFK5 504304 1:20
Thermo Fisher IL-17A Monoclonal Antibody (eBio17B7), eFluor 506 eBio17B7 69-7177-82 1:20
Biolegend APC/Cyanine7 anti-mouse IFN-γ Antibody XMG1.2 505850 1:20
Thermo Fisher PE anti-mouse IL-13 eBio13A 12-7133-82 1:20

Human antibodies
Vendor Antibody Clone Catalog Number Dilution
Miltenyi FcR Blocking Reagent, human 130-059-901 1:50
Biolegend FITC anti-human CD3 Antibody UCHT1 300440
Biolegend FITC anti-human CD4 Antibody OKT4 317408
Biolegend FITC anti-human CD8a Antibody RPA-T8 301050
Biolegend FITC anti-human CD14 Antibody 63D3 367116
Biolegend FITC anti-human CD15 (SSEA-1) Antibody HI98 301904
Biolegend FITC anti-human CD16 Antibody 3G8 302006
Biolegend FITC anti-human CD19 Antibody HIB19 302206
Biolegend FITC anti-human CD20 Antibody 2H7 302304
Biolegend FITC anti-human CD33 Antibody HIM3-4 303304
Biolegend FITC anti-human CD34 Antibody 561 343604
Biolegend FITC anti-human CD203c (E-NPP3) Antibody NP4D6 324614
Biolegend FITC anti-human FcεRIα Antibody AER-37 (CRA-1) 334608
Biolegend Brilliant Violet 650™ anti-human CD335 (NKp46) Antibody 9E2. 331927 1:50
Biolegend Brilliant Violet 421™ anti-human CD294 (CRTH2) Antibody BM16 350112 1:50
Biolegend PE/Cyanine5 anti-human CD117 (c-kit) Antibody A3C6E2 323412 1:25
Biolegend APC anti-human CD127 (IL-7Rα) Antibody A019D5 351316 1:50
Biolegend APC/Cyanine7 anti-human CD45 Antibody 2D1 368516 1:50

Validation

All antibodies were validated based on company website product data sheets

Animals and other organisms

Policy information about studies involving animals; ARRIVE guidelines recommended for reporting animal research

Laboratory animals

Strain/Source/Age/Sex
C57BL/6J (Wild-type) Jackson Labs 6-12 weeks Female mice were used in all experiments except 10-day Alternaria-challenge experiments (used both male and female mice)
Rbm3-/- from Peter Vanderklish 6-12 weeks Female mice were used in all experiments except 10-day Alternaria-challenge experiments (used both male and female mice)
Tslpr-/- from Michael Croft 6-8 weeks Female mice
Rbm3-/-Rag2-/- Crossed in-house 6-15 weeks Male mice
Rag2-/- from Jackson Labs 6-15 weeks Male mice
Rbm3-/-Cysltr1-/- Crossed in-house 8-10 weeks Female mice
Cysltr1-/- from Jackson Labs 8-10 weeks Female mice
CD45.1+ Pep Boy Homo. Jackson Labs 18 weeks Female mice
CD45.1+CD45.2+ Pep Boy Het. Jackson Labs 18-20 weeks Female mice

Wild animals

No wild animals

Field-collected samples

No field collected samples

Ethics oversight

UCSD IACUC approved all studies

Note that full information on the approval of the study protocol must also be provided in the manuscript.

Flow Cytometry

Plots

Confirm that:

- [x] The axis labels state the marker and fluorochrome used (e.g. CD4-FITC).
- [x] The axis scales are clearly visible. Include numbers along axes only for bottom left plot of group (a 'group' is an analysis of identical markers).
- [x] All plots are contour plots with outliers or pseudocolor plots.
- [x] A numerical value for number of cells or percentage (with statistics) is provided.

Methodology

Sample preparation

For surface stains, 1x106 lung and BAL cells were stained. For intracellular stains, 2 x 106 lung cells were stained. Fc receptors were first blocked for 5 minutes using CD16/CD32 (Biolegend, San Diego, CA). Eosinophils were identified as CD11cSiglec-F

+ and neutrophils were identified as SiglecFGR-1+; they were stained using CD45.2 (PerCP), Siglec-F (PE), GR-1(APC), and CD11c (FITC). ILCs were identified as LineageThy1.2+ lymphocytes or LineageT1ST2+ lymphocytes and were stained using CD45.2 (PerCP), Thy1.2 (APC), T1ST2 (PE), and a lineage cocktail. The lineage cocktail (FITC) consisted of the BioLegend Lineage cocktail (consists of CD3e, Ly-6G/Ly-6C, CD11b, CD45R/B220, and TER-119), CD11c, NK1.1, CD5, FcεR1, TCR , and TCRɣδ. The ILC subsets were stained with ST2 (APC) and CD127 (PE-Cy7). For nuclear intracellular staining, cells were permeabilized using the FoxP3 kit (ThermoFisher, Waltham, MA) after surface staining. Cells were stained with Ki-67 (PE or APC), RBM3, GATA3 (PE), and ID2 (PE).

For cytokine intracellular staining of lung cells in the 10-day challenge model, cells were cultured overnight with Golgi Plug (Fisher Scientific, Hampton, NH) at 500,000 cells per well. After surface staining for ILCs, cells were fixed and permeabilized using the BD fixation/permeabilization kit (BD Biosciences, La Jolla, CA) and stained for IL-5 (PE) or IL-13 (PE). For cytokine intracellular staining following the 7-day IL-33 and Alternaria challenge model, lung cells were cultured for 3 hours with cell stimulation cocktail (ThermoFisher, Waltham, MA) at 1x106 cells per well. After surface staining for ILCs, cells were fixed and permeabilized using the BD kit and stained for IL-5 (PE), IL-13 (PE), or IL17A (eFlour506). Lung cells stained for Bcl-2 expression were surfaced stained for ILCs, fixed and permeabilized with the BD kit, and stained with anti-Bcl-2 (PE-Cy7).

For human PBMC staining, ILC2s were sorted as LineageCRTH2+ lymphocytes. The lineage cocktail (FITC) consisted of antibodies for CD3, CD14, CD16, CD19, CD20, CD56, TCRγδ, CD4, CD11b, CD235a, and FcεRI. The polyclonal RBM3 antibody used in this study was raised in rabbits to the 14 c-terminal amino acids of RBM3, and affinity purified to the immunizing peptide. As described in prior work65, 66, the affinity purified anti-RBM3 antibody recognizes an ~17 kDa band corresponding to RBM3 on Western blots and selectively labels RBM3 in situ under a variety of fixation conditions. Flow Cytometry was performed using the BD Accuri for Fig. 1 and Supplemenatary Fig. 3b, otherwise Acea Novocyte was used. Data was analyzed using FlowJo software (Tree Star, Ashland, OR). All antibodies were from Biolegend, ThermoFisher, or BD Biosciences.

Instrument	Acea Novocyte and BD Accuri
Software	Data was analyzed using FlowJo software (Tree Star, Ashland, OR).
Cell population abundance	1,000,000 cells per tube were collected.
Gating strategy	All parent gating began with live gates, FSC/SSC, followed by either lymphocyte or granulocyte gates as shown in primary figures and supplemental methods. ILCs were then gated as lineage-negative thy1.2+. Human ILC2s gated the same but with final gate as CRTH2+. Sort purities were >99% as shown in Supplmentary Figure 9.

☒ Tick this box to confirm that a figure exemplifying the gating strategy is provided in the Supplementary Information.
